# Spatial variations in vegetation fires and emissions in South and Southeast Asia during COVID-19 and pre-pandemic

**DOI:** 10.1038/s41598-022-22834-5

**Published:** 2022-10-29

**Authors:** Krishna Vadrevu, Aditya Eaturu, Emily Casadaban, Kristofer Lasko, Wilfrid Schroeder, Sumalika Biswas, Louis Giglio, Chris Justice

**Affiliations:** 1 NASA Marshall Space Flight Center, Huntsville, Alabama 35811 USA; 2 University of Alabama in Huntsville, Huntsville, AL USA; 3 Geospatial Research Laboratory, Engineer Research and Development Center, U.S. Army Corps of Engineers, Alexandria, VA USA; 4 NOAA NESDIS, College Park, Maryland 20740 USA; 5 University of California Los Angeles, Los Angeles, USA; 6 University of Maryland, College Park, Maryland 20742 USA

**Keywords:** Environmental sciences, Environmental chemistry, Environmental impact

## Abstract

Vegetation fires are common in South/Southeast Asian (SA/SEA) countries. However, very few studies focused on vegetation fires and the changes during the COVID as compared to pre-pandemic. This study fills an information gap and reports total fire incidences, total burnt area, type of vegetation burnt, and total particulate matter emission variations in SA/SEA during COVID-2020 and pre-pandemic (2012–2019). Results from the short-term 2020-COVID versus 2019-non-COVID year showed a decline in fire counts varying from − 2.88 to 79.43% in S/SEA. The exceptions in South Asia include Afghanistan and Sri Lanka, with a 152% and 4.9% increase, and Cambodia and Myanmar in Southeast Asia, with an 11.1% and 8.5% increase in fire counts in the 2020-COVID year. The burnt area decline for 2020 compared to 2019 varied from − 0.8% to 92% for South/Southeast Asian countries, with most burning in agricultural landscapes than forests. Several patches in S/SEA showed a decrease in fires for the 2020 pandemic year compared to long term 2012–2020 pre-pandemic record, with Z scores greater or less than two denoting statistical significance. However, on a country scale, the results were not statistically significant in both S/SEA, with Z scores ranging from − 0.24 to − 1, although most countries experienced a decrease in fire counts. The associated mean TPM emissions declined from ~ 2.31 Tg (0.73stdev) during 2012–2019 to 2.0 (0.65stdev)Tg in 2020 in South Asia and 6.83 (0.70stdev)Tg during 2012–2019 to 5.71 (0.69 stdev)Tg in 2020 for South East Asian countries. The study highlights variations in fires and emissions useful for fire management and mitigation.

## Introduction

Vegetation fires are a recurrent phenomenon in several ecosystems of South/Southeast Asia (SA/SEA). Fires can determine the type of vegetation and composition and alter the landscape's structure^1^ and ecological processes^2^. In particular, tropical ecosystems in Asia are dominated by dry deciduous, thorn, and mixed deciduous forests, and fire is considered the most common disturbance due to the increasing dependence of people on these forests^3^. The impact of fires on landscape structure and composition in different world ecosystems is well-documented^4^. The consequences can be both positive and negative, depending on the intensity of fires, the level of fire adaptation of the ecosystem, and the affected landscape. The positive effects include facilitating plant nutrient uptake, promoting new grass cover useful to herbivores in some ecosystems^5^ and reducing the fuel loads and the intensity of fires in subsequent burns. Specific to the adverse effects, fires result in a loss of vegetation and impact ecosystem services, such as timber, shelter, nutrients, and water retention, including recreation^6,7^. Repeated burning also modifies the nutrient balance of soils, primarily through pyro-denitrification^8^. The drivers of fires can include both climate and anthropogenic factors^9,10^. In several SA/SEA countries, fires are used as a management tool for clearing forests through slash and burn in several regions such as Dhading and Chitwan districts, Nepal^11^; the Eastern Ghats^12^ and northeast India^13^; Chittagong hill tracts of Bangladesh^14^; Bago mountains, Myanmar and Shan state, Myanmar^15^; Sarawak in Malaysia^16^; Caraballo mountain in Carranglan and Mount Mingan, Philippines^17^; Jambi Province, Sumatra, and others in Indonesia^18^; northern Thailand^19^; north-western Cambodia^20^; northern Laos^21^ and northern Vietnam^22^. Fires are also extensively used for clearing land for rubber and oil palm expansion in Indonesia^23^. In addition, to slash and burn, most of the countries in S/SEA are agrarian, where farmers burn agricultural residues to clear the land for the next crop, such as India, Pakistan, Myanmar, Thailand, and Vietnam. The residue burning practices vary in different countries, such as burning residues on the ground surface after harvest or collecting and piling the residues followed by burning^23^. These varied management practices and local land use policies^24^, including natural variations in climate^24,25^, can cause inter-annual variability in fires and satellite fire detections^10^. The biomass burning resulting from such activities is an important source of greenhouse gas emissions and aerosols^8^. These aerosols can significantly impact air quality at local and regional scales^26^.

Information on fire occurrences and their timing, including spatial and geographic gradients, can help understand the impacts of fires, such as on changes in landscape structure and function, including air pollution for improved fire management^27,28^. Over the past decades, spatial information technologies have been widely used in fire detection, mapping, and monitoring studies. In particular, remote sensing technology with its multitemporal, multispectral, synoptic, and repetitive coverage capabilities can provide valuable information on the fire occurrence, intensity, the amount of area burned and the type of vegetation impacted^10^. Because of their high temperature, fires emit thermal radiation with a peak in the middle infrared region, following Planck's theory of blackbody radiation. Therefore, active fire sensing is often done using mid-infrared and thermal infrared (usually around 3.7 to 11 μm) information from satellites^29^. The most commonly available remote sensing global fire datasets are from the Moderate Resolution Imaging Spectroradiometer (MODIS) onboard NASA’s Terra and Aqua satellites and the Visible Infrared Imaging Radiometer Suite (VIIRS) on the Joint Polar Satellite System (JPSS). The MODIS and VIIRS active fire global products are available at 1 km and 375 m resolution, respectively^30,31^. These fire datasets can be used to address fire management and mitigation efforts at different scales.

In this study, we addressed specific questions related to vegetation fire characteristics in SA/SEA region during the COVID-19 pandemic compared to recent pre-pandemic years. COVID-19 was first reported in December 2019 in Wuhan, Hubei Province, China^32^. Since then, COVID has been reported throughout the globe, reaching pandemic levels. Information on COVID spread, deaths, impacts on the economy, lockdowns, etc., in South/Southeast Asia were reported in various papers^32–36^. During the COVID-2020 year, due to the reduced use of fossil fuels, several studies also reported lower pollution levels, including those in SA/SEA^36–39^. In the early stages of the outbreak, attempts were made to trace every infection back to its origin. However, tracing back to the origin of COVID cases at a local, regional, and international level soon became impossible; thus, most countries responded by imposing lockdowns (see Table 1 supplementary materials). These lockdowns aimed to slow down the pandemic by restricting mobility so that the disease doesn't spread to new places. Mainly, the mobility of migrant workers, laborers, and the public was severely restricted. In particular, many migrant workers returned home from cities to rural villages in several countries, with more dependence on natural resources at their local places^35^. As a result, a significant reduction in fossil fuel emissions during the pandemic was reported in different cities^36–38^; however, it is unclear whether vegetation fires and emissions were equally affected due to the mobility restrictions, which is the focus of this study.

Specifically, we addressed the following questions: a). How did the total number of fires vary during the COVID-2020 year versus the previous non-COVID year 2019 and pre-pandemic years (2012–2019) in SA/SEA countries? How did the fire-related total particulate matter (TPM) emissions vary during COVID-2020 and in previous years? We addressed these questions using fire data from the SNPP-VIIRS (2012–2020) and MODIS (2000–2020) satellites in SA/SEA countries. We hypothesized that the fire activity during the 2020 COVID year changed as compared to pre-pandemic years, probably due to changes in human mobility due to COVID lockdowns, and the changes vary spatially in different countries. This hypothesis was tested for fire variations, and at multiple spatial scales from grids to regional means. The results highlight variations in fires and TPM emissions during COVID-2020 versus previous years.

### Datasets

#### VIIRS fire counts

**Figure 1:**
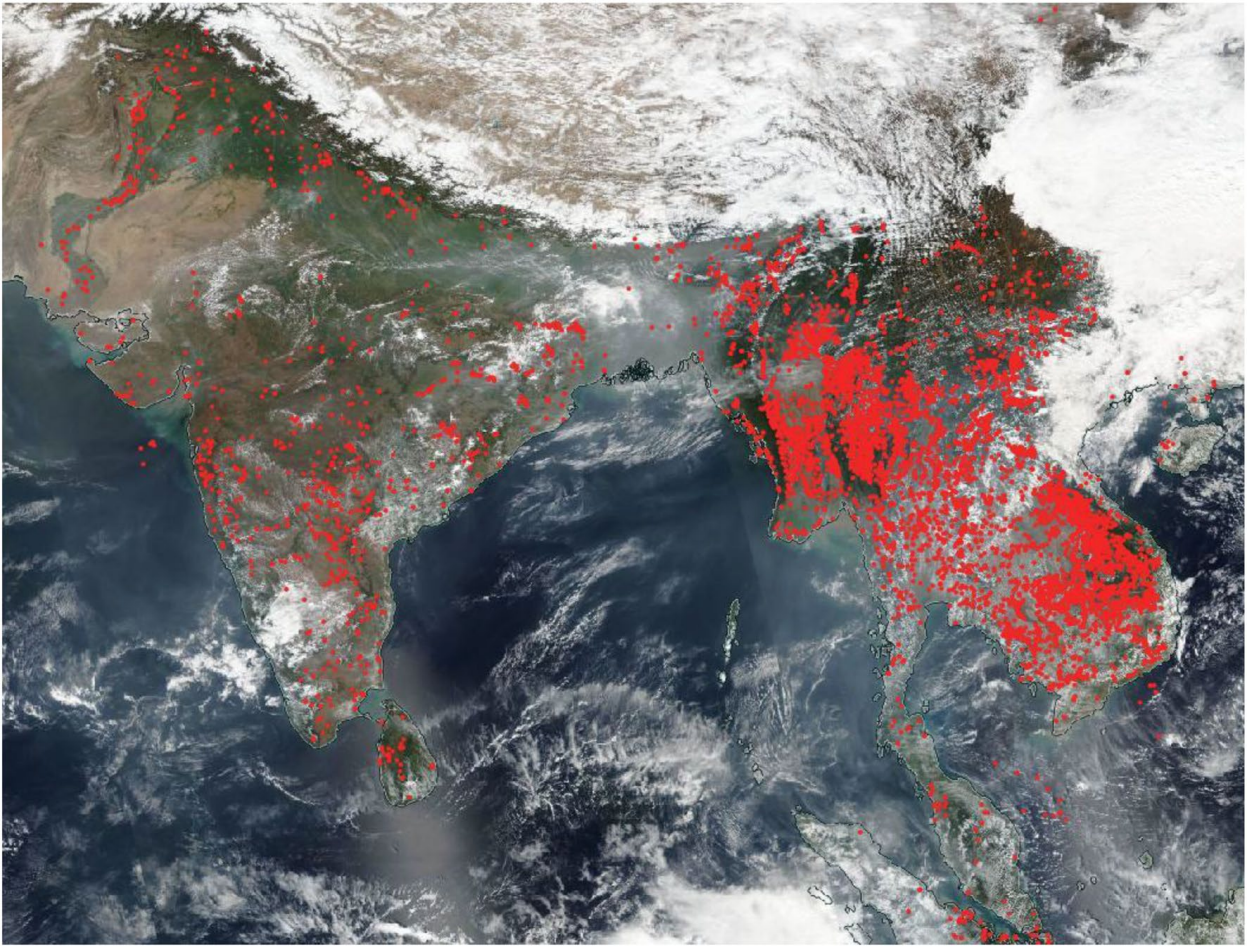
Suomi NPP/VIIRS fires and thermal anomalies shown as red dots (Day and Night) on March 02nd, 2020. The background image is NOAA-20/VIIRS corrected reflectance with true color (Red = Band I1; Green = Band M4 and Blue = Band M3). The VIIRS instrument is aboard the joint NASA/NOAA NOAA-20 (JPSS-1) satellite. (Imaged generated using opensource free NASA WorldView system (https://worldview.earthdata.nasa.gov/).

To address the fire variations in different countries during and pre-pandemic, we used the 375 m active fire product derived from the VIIRS instruments onboard the Suomi National Polar-orbiting Partnership (S-NPP) and NOAA-20 satellites. In contrast to other coarser resolution satellite fire detection products such as MODIS (≥ 1 km), the improved 375 m data provide increased detection of smaller fires and enhanced mapping of large fire perimeters^31^. The VIIRS 375 m fire product builds on the earlier MODIS fire product heritage^40–42^, using a multispectral contextual algorithm to identify sub-pixel fire activity and other thermal anomalies in the Level 1 (swath) input data. The algorithm uses all five 375 m VIIRS channels to detect fires and separate land, water, and cloud pixels in the image^31^. In the VIIRS data, for the daytime data, cloud pixels are classified using brightness temperature (BT) tests in channel 5 (< 265 K) or reflectance in I-band channel ρ1 + ρ2 > 0.9 and BT_5_ < 295 K or ρ1 + ρ2 > 0.7 and BT_5_ < 285 K where ρ_i_ is the reflectance in I-band channel I and BT_i_ is the brightness temperature in I-band channel *i*). For nighttime data, cloud pixels are classified based on the brightness temperature of channels I4 and I5 as BT_5_ < 265 K and BT_4_ < 295 K. Using these tests, the fire algorithm skips all day and nighttime pixels classified as cloud-covered, and their data are excluded from the calculation of fire pixel background conditions. This is a typical limitation of any optical remote sensing data where clouds can be a persistent problem hindering land surface (or fire) retrievals. For example, in Southeast Asia, clouds or thick haze during the biomass burning months, i.e., July–October of every year, can result in significant variations impacting satellite fire detections^31^. A more detailed study is needed to study such variations. Near real-time data are available in various formats, including the TXT, SHP, KML, and WMS from https://earthdata.nasa.gov/active-fire-data. Figure 1 depicts the Suomi NPP/VIIRS fires data on March 02nd, 2020. A decrease in the Sum of FRP in any specific year indicates reduced biomass consumption and thus reduced emissions to the atmosphere. Besides, we also used the FRP data to derive Total Particulate Matter (TPM) emissions during 2020 and previous years. We also calculated percent differences between the TPM during 2020 versus 2019 and the earlier period (2012–2019).

#### MODIS burnt areas

To assess the burnt area variations, we used the latest MCD64A1 burned area product (Collection 6), covering different countries and periods during and pre-pandemic. The product is generated using an improved version of the MCD64 burned area mapping algorithm^42^ based on the Collection-6 surface reflectance and active fire input data. The MODIS burned area mapping algorithm takes advantage of spectral changes resulting from the alteration of vegetation structure or vegetation removal and deposits of charcoal and ash, which might vary based on the site conditions. The burned area product integrates both 500 m MODIS imagery coupled with 1 km MODIS active fire observations. The hybrid algorithm applies dynamic thresholds to composite imagery generated from a burn-sensitive vegetation index (VI) derived from MODIS short-wave infrared channels 5 and 7 and a temporal texture measure^43^. As part of the process, cumulative active fire maps are used to filter the burned and unburned areas and to guide specific prior probabilities. The combined use of active-fire and reflectance data enables the algorithm to adapt regionally over a wide range of pre-and post-burn conditions and across multiple ecosystems. The product contains five data layers (Burn Date, Burn Date Uncertainty, QA, First Day, and Last Day), each stored as a separate HDF4 Scientific Data Set (SDS). In this study, we used a quality filter to ensure that land only pixels were included in BA (QA-bit 0) and sufficient valid data in the reflectance time series for the processed grid cell (QA-bit 1). More details about the product can be found in the Collection 6 MODIS Burned Area Product User's Guide (v.1.3, 2020), available at https://modis-fire.umd.edu/files/MODIS_C6_BA_User_Guide_1.3.pdf.

## Methods

### Inter-annual variations in vegetation fires

Using the VIIRS fire count (FC) datasets, we computed the year-to-year variations to infer if vegetation fires decreased or increased during the COVID-2020 year associated with the initial phase of the pandemic. We assessed the fire data at two different scales, first using a 30-min grid (0.5°) across SA/SEA and second at the individual country level.

For the spatial analysis, we first gridded the daily VIIRS fire data at 0.5° for individual months and years (2012–2020). The monthly fire data (M) were given as,

1 $$M_{k, i } = \mathop \sum \limits_{d = 1}^{n} N_{k, d }$$

where *k* represents the 0.5° grid cells, *i* represent the month, ${N}_{k, d }$ represents fire counts data value for each day *d*, for each calendar month *i*.

The yearly FC data ${Y}_{k, l}$ for each grid cell *k* and year *l* is given as

2 $$Y_{k,l } = \mathop \sum \limits_{i = 1}^{12} M_{k,i }$$

For each 0.5° grid cell (*k*), we calculated the relative change (%) ${Y}_{c}$ in fire counts during 2020-COVID year compared to 2019 non-COVID year as,

3 $$Y_{c } = \frac{{Y_{k, 2020} - Y_{k, 2019 } }}{{Y_{k, 2019} }} \times 100$$

We also compared the 2020-COVID year fire counts with the previous years (2012–2019) mean fire FC in two different steps:

- (i). The mean annual FC averaged between 2012 and 2019 denoted as *Avg* for each grid cell *k* and year *l* is calculated as,
  4 $$Avg_{k, l} = \frac{{\mathop \sum \nolimits_{l = 1}^{8} \;Y_{k, l} }}{{T_{k, l} }}$$
  where, *T* represents the total number of years spanning 2012 to 2019.
- (ii). The percent change ${P}$ for each grid cell *k* and year *l* in FC between the year 2020 versus the *Avg* during previous years (2012–2019) is given as,
  5 $$P_{k,l } = \frac{{Y_{k, 2020} - Avg_{k, l} }}{{Avg_{k, l} }} \times 100$$
  In the results section, we report the inter-annual variations in FC for both gridded and country-specific data.

### Spatial and temporal fire variations

In addition to addressing the percent change in fire counts described above, we also used the Z-score statistic to infer the fire count variations specific to the pandemic year 2020 compared to the entire fire study period (2012–2020). The Z-score tells how many standard deviations away a value is from the mean and is computed as:

$$z = \left( {X{-}\mu } \right)/\sigma$$

where, *X* is the fires in the pandemic year 2020, *μ* is the mean fire counts from 2012 to 2020, and *σ* is the standard deviation in the fire datasets. Z-scores may be positive or negative, with a positive value indicating the score above the mean and a negative score below the mean. Thus, if a Z-score is 0, the data point's score is identical to the mean score. A Z-score of 1.0 indicates a value of one standard deviation from the mean, and − 1 indicates below the mean, etc. A departure from the mean by more than two standard deviations is usually considered as significantly different. The results are reported for the individual countries and at a 30-min grid scale.

### Emissions variation between COVID-2020 versus previous years

We used the VIIRS derived fire radiative power (FRP) to infer TPM variations during 2020 and previous years. The FRP is the rate of fire energy released per unit time and measured in megawatts^44,45^. The satellite-based FRP was derived by^44^ as,

6 $$FRP \approx \frac{{A_{pix} \sigma }}{{a \tau_{4} }}\left( {L_{4} - \underline {L}_{4} } \right)$$

where, *L*_4_ is the 4 μm radiance of the fire pixel, ${\underline{L}}_{4}$ is the 4 μm background radiance, *A*_*pix*_ is the area of the pixel (which varies as a function of scan angle), *σ* is the Stefan-Boltzmann constant (5.6704 × 10^−8^ W m^−2^ K^−4^), ${\tau }_{4 }$ is the atmospheric transmittance of the 4 μm channel, and *a* is a sensor-specific empirical constant.

The FRP measurements have been previously related to the amount of biomass burnt^45,46^, the strength of fires^46^ and aerosol emissions^47–51^ . FRP integrated over space and time results in Fire Radiative Energy (FRE) with units in megajoules. In general, the biomass burning emissions are estimated using the FRE after^56–58^,

7 $$E = DM \times F = FRE \times \beta \times F = \int_{{t_{1} }}^{{t_{2} }} {FRPdt \times \beta \times F}$$

In the above equation, *E* represents the emissions, *DM* is the dry fuel mass combusted (kg), *F* is the fraction of consumed biomass that is released as emissions, *FRE* is the Fire Radiative Energy (MJ), *t1* and *t2* are the beginning and end timing of the fire events (seconds), $\beta$ is the biomass combustion rate (Kg/MJ).

The biomass combustion rate of 0.368–0.015 (kg/MJ) was derived by^45^ based on field and controlled experiments regardless of the land surface conditions, and several researchers have used these coefficients for calculating the satellite-based fire-related emissions^52–54^. Also, ^44,55^, developed the FRE-based emission coefficients for quantifying the gas and aerosol emissions from biomass burning. In later developments, the intermediate step of quantifying dry matter combustion rate (kg s^−1^) was bypassed by deriving smoke emission coefficient (*C*e) rates which describe the relationship between the FRE (MJ) and the mass of TPM (in kg or g) it emits, or between the rates of these two (i.e. the FRP in MW and the TPM emission rate in g.s^−1^) and *C*e has units of g.MJ^−1^ or g.s^−1^.MW^−1^ respectively, derived from a set of matchup fires for which good observations of both variables exist^56^. Thus, for example^56^, related the rate of aerosol emission (Rsa in kg/sec) to FRP as,

8 $$R_{sa} = C_{e} \times FRP$$

where, *C*_*e*_ is the coefficient that directly relates radiative power from fire to its smoke aerosol emission rate (coefficients of emission in kg/MJ for particulate matter) and Mass of smoke aerosol emission (*M*_*sa*_) as,

9 $$M_{sa} = C_{e} \times FRE$$

Using the above approach^57^, developed a global emissions inventory product titled Fire Energetics and Emissions Research (FEER) v1 based on collocated satellite FRP and MODIS Dark Target (DT) aerosol optical thickness (AOT) observations. Inferred TPM emissions rates are linked to observed FRP. The estimated TPM emission coefficients allow direct conversion from time-integrated FRP to emitted particulate matter without invoking the emissions factors. An improvement to the above approach was from^58^, who used a MODIS Deep Blue (DB) AOT product to develop biome-dependent Fire Radiative Energy Emissions (FREMv1), since the DB product showed better agreement with AERONET AOT observations across southern Africa than the DT product. Both the DB and DT AOT products that are used in FEER and FREMv1 are at 10 km resolution. Recently^59^, used the Multiangle Implementation of Atmospheric Correction (MAIAC) algorithm, derived 1 km AOT from refining the FREMv.1 and developed the FREMv.2 product over the African continent, covering different biomes with improved smoke emission coefficients (Ce in g MJ^−1^). The estimated TPM emission coefficients from both the FEERv1 and FREMv2 approaches allow direct conversion from FRP to emitted particulate matter without invoking the emission factors. In this study, we preferred FEER coefficients as the product involved FRP-AOT match-up cases from Asia^57^.

## Results

### Country level variations in fires

**Figure 2:**
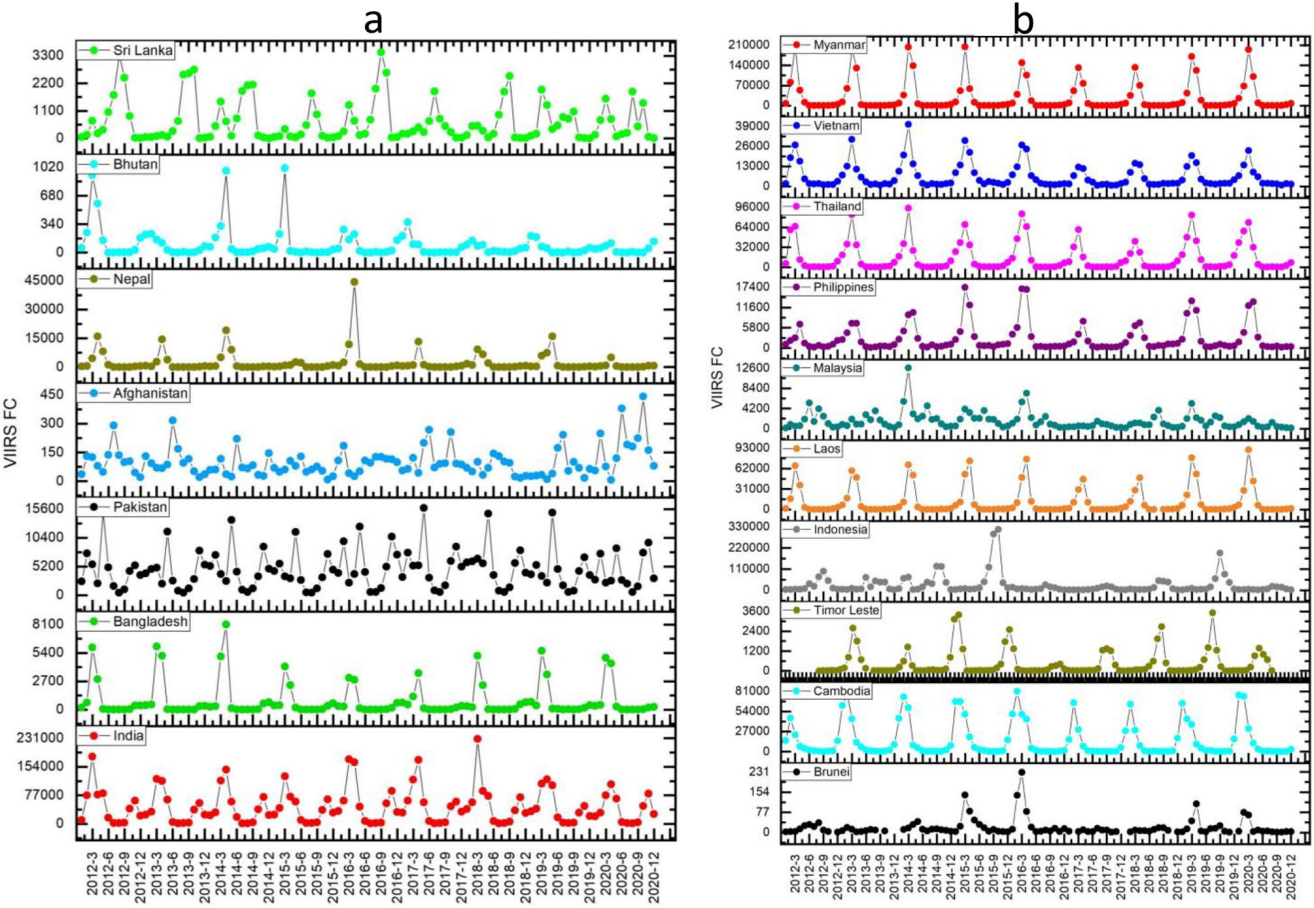
(**a**), (**b**) VIIRS (375 m) retrieved fire counts (FC) for South and Southeast Asian countries.

Variations in VIIRS derived fire counts (FC) for different SA/SEA countries are shown in Fig. 2a,b. MODIS and VIIRS annual fire detections for different years in SA/SEA countries are shown in Table 1a,b. On average, VIIRS could detect 5.28 times more fires in South Asia and 5.12 times more fires in Southeast Asian countries. In South Asia, India had the highest FC (*µ* = 555,651; *SD* = 71,222.0), followed by Pakistan (*µ* = 55,670.3; *SD* = 6031.7), Nepal, Bangladesh, Sri Lanka, Bhutan, and least in Afghanistan (µ = 1063; SD = 433.9). In the case of Southeast Asian countries, Myanmar had the highest FC (*µ* = 347,930; *SD* = 54,111.2), followed by Indonesia (*µ* = 316,753; *SD* = 254,271), Thailand, Cambodia, Laos, Vietnam, Philippines, Malaysia, Timor Leste and the least in Brunei (*µ* = 209; *SD* = 110.5). At a country level in SA, the coefficient of variation (CV) in FC (2012–2020) was relatively high for Nepal (*CV* = 0.59), Bhutan (0.54), Sri Lanka (0.32), Bangladesh (0.27), Afghanistan (0.20), India (0.12) and least for Pakistan (0.11). In SEA, the highest CV was found for East Timor (141.9), followed by the Philippines (114.17), Brunei (109.0), Singapore (108.31), Cambodia (103.50), Malaysia (94.29), Myanmar (87.51), Indonesia (86.21), Laos (80.37), Thailand (4846) and least for Vietnam (25.33).

**Table 1:** a, b. MODIS and VIIRS annual fire detections for different years in South/Southeast Asian countries. MODIS detections shown in parenthesis and VIIRS without. On an average VIIRS could detect 5.28 times more fires in South Asia and 5.12 times more fires in Southeast Asian countries.

Country	2012	2013	2014	2015	2016	2017	2018	2019	2020	VIIRS fire detections higher than MODIS
**a**										
Afghanistan	(220) 1251	(216) 1253	(185) 944	(112) 764	(188) 1195	(228) 1441	(115) 864	(158) 856	(435) 2164	5.78
Bangladesh	(3251) 10,673	(2933) 12,799	(2818) 15,315	(1588) 8548	(1852) 7535	(1468) 6630	(2289) 9375	(2719) 10,966	(2903) 11,054	4.26
Bhutan	(508) 2168	(274) 849	(418) 1774	(302) 1364	(264) 901	(274) 849	(168) 587	(155) 605	(154) 478	3.80
India	(93,150) 579,469	(71,173) 489,144	(76,416) 536,157	(68,433) 479,234	(88,809) 674,484	(82,545) 596,994	(91,110) 642,983	(75,502) 531,727	(76,021) 470,667	6.92
Nepal	(3581) 31,490	(2534) 22,665	(3443) 35,244	(1083) 8669	(7767) 62,025	(2218) 17,721	(2990) 21,996	(3950) 31,299	(1352) 8409	8.28
Pakistan	(12,133) 54,595	(9054) 48,845	(10,255) 56,487	(8610) 48,118	(10,853) 61,945	(12,234) 63,922	(12,961) 63,607	(9578) 52,647	(9904) 50,865	5.24
Sri Lanka	(1821) 10,926	(1137) 9289	(1371) 9901	(669) 4232	(1468) 11,587	(719) 5491	(938) 7007	(975) 7185	(736) 7534	7.44
**b**										
Brunei	(64) 152	(61) 76	(70) 169	(101) 374	(122) 540	(26) 63	(15) 87	(58) 240	(34) 183	3.42
Cambodia	(30,514) 113,223	(44,938) 217,328	(39,916) 202,624	(44,023) 231,456	(37,153) 233,615	(22,426) 125,834	(28,815) 149,825	(32,822) 180,640	(32,926) 200,712	5.28
East Timor	(838) 6352	(318) 2632	(1171) 8966	(740) 6400	(171) 1252	(560) 4342	(753) 6166	(956) 7420	(455) 4217	8.01
Indonesia	(71,135) 309,045	(53,852) 251,218	(115,121) 529,883	(178,578) 860,671	(24,061) 112,502	(17,823) 87,054	(38,074) 185,288	(90,475) 427,265	(16,201) 87,853	4.71
Laos	(40,099) 128,134	(39,920) 137,856	(42,428) 140,502	(41,919) 153,870	(42,147) 151,423	(27,366) 101,626	(28,094) 102,191	(49,073) 172,356	(47,330) 177,161	3.53
Malaysia	(5602) 19,339	(4539) 16,970	(8468) 37,399	(5821) 23,698	(5715) 25,027	(2177) 7337	(3376) 13,274	(4735) 19,973	(2672) 9333	4.00
Myanmar	(80,799) 362,474	(69,719) 403,292	(67,995) 395,333	(58,367) 341,781	(51,514) 322,003	(47,371) 279,117	(42,459) 254,106	(59,067) 370,347	(66,988) 402,919	5.75
Philippines	(3271) 19,793	(4158) 25,949	(5392) 34,169	(7511) 45,575	(7271) 49,782	(3367) 19,108	(4718) 27,496	(6886) 37,398	(5941) 33,085	6.23
Singapore	(7) 35	(9) 28	(9) 31	(6) 55	(7) 19	(8) 25	(5) 28	(4) 25	(1) 15	4.66
Thailand	(30,913) 160,504	(31,495) 193,562	(31,008) 196,181	(27,880) 187,428	(30,029) 233,645	(18,563) 130,490	(18,089) 114,742	(34,697) 232,841	(30,233) 217,462	6.59
Vietnam	(20,683) 80,176	(17,745) 80,799	(23,766) 102,652	(22,672) 100,107	(21,133) 90,907	(11,893) 45,851	(13,366) 57,132	(18,588) 72,267	(17,820) 70,178	4.18

**Figure 3:**
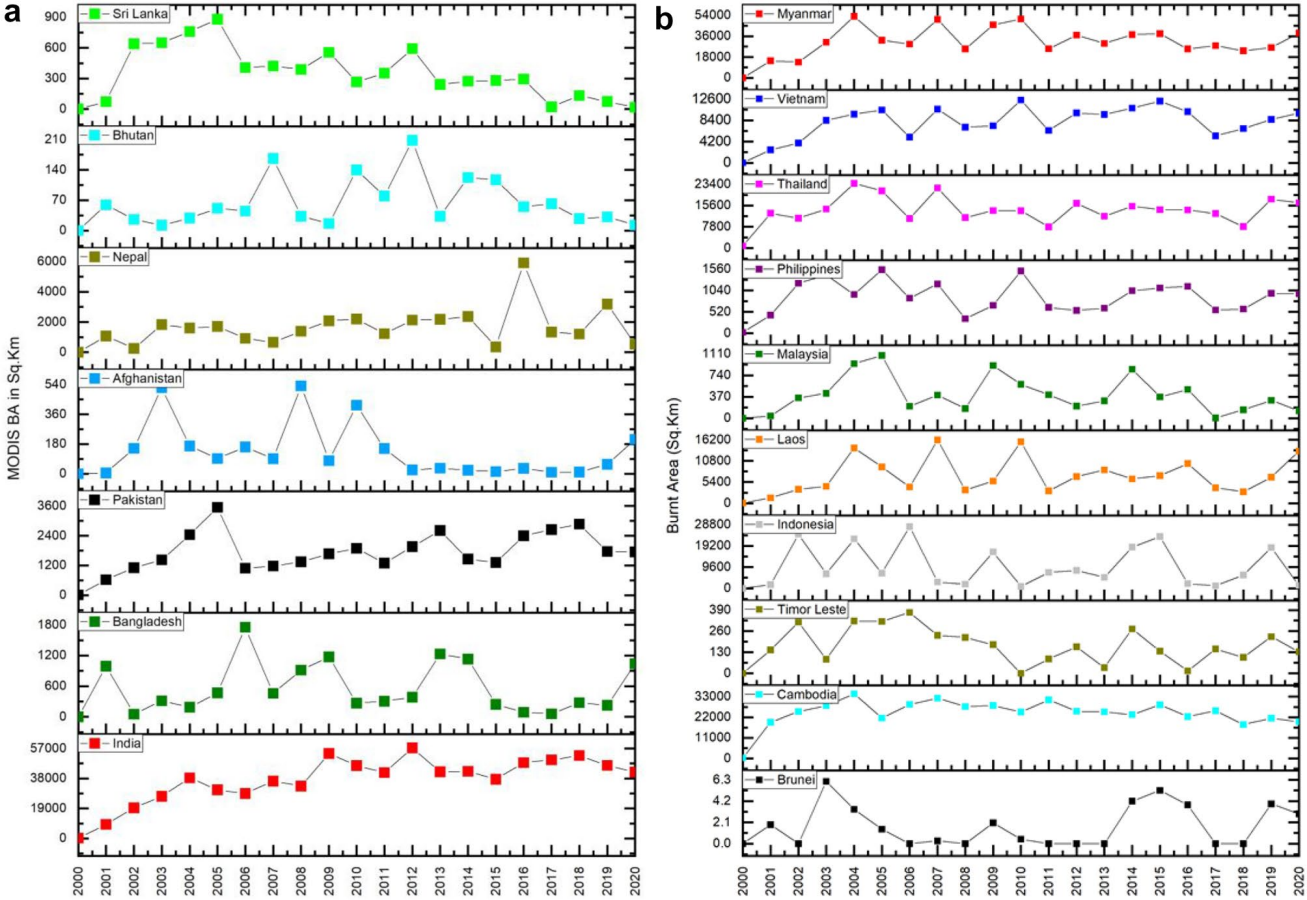
(**a**), (**b**). Burnt areas (in sq.km) for different countries in South Asia (10a) and Southeast Asia (10b).

The burnt areas (BA's in sq. km) inter-annual variability derived from MODIS MCD64A1 (500 m) product for different SA/SEA Asian countries from 2000–2020 are given in Fig. 3a,b. In South Asia, India had the highest BA (*µ* = 37,226; *SD* = 14,470.1), followed by Pakistan (*µ* = 1733; *SD* = 811.5), Nepal, Bangladesh, Sri Lanka, Afghanistan, and least in Bhutan (*µ* = 63; *SD* = 55.9). In the case of SEA countries, Myanmar had the highest BA (*µ* = 31,204; *SD* = 13,033.4), followed by Cambodia (*µ* = 24,348; *SD* = 7010.3), Thailand, Indonesia, Vietnam, Laos, Philippines, Malaysia, Timor Leste, and least in Brunei (*µ* = 1.70;*SD* = 2.0).

In addition to the total BA for individual countries, inter-annual variability in BA in different land cover classes from 2000–2020 are also shown for different countries in South Asia and Southeast Asia (Supplementary materials Figs. 1a,b,c,d,e,f,g and 2a,b,c,d,e,f,g,h,i,j). A closer look at the BA in South Asian countries suggests a relatively higher percentage in croplands for India (68.8%), Pakistan (96%), and Sri Lanka (88%) compared to forest fires in Bhutan (82%) and Nepal (84%). Also, an apparent increase in cropland fires can be seen from 2000–2020 in India and Pakistan compared to Sri Lanka. In SEA countries, Myanmar (62.7%) and Cambodia (73.4%) had the most burnt areas in forests, whereas the rest of the countries, i.e., Laos, Indonesia, Thailand, Vietnam, Malaysia, etc., had the most burnt areas in croplands.

### Fire variations Pre-COVID and COVID year

**Figure 4:**
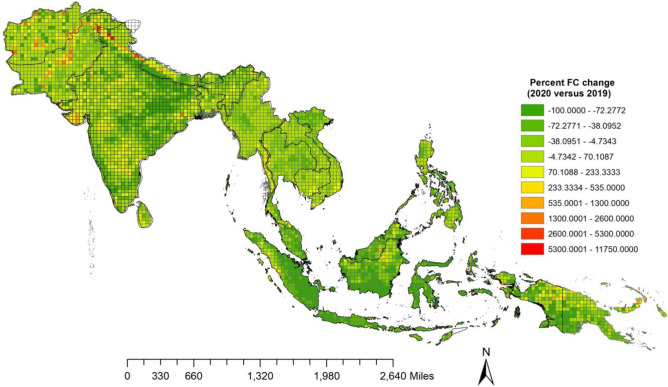
Percent FC change during 2020 versus 2019 for South/Southeast Asia. VIIRS I-band (375 m) fire counts were spatially gridded at 30 min intervals (0.5 × 0.5degree cells) for the regional scale analysis. A clear percent increase in fires can be seen in northern Himalayan region, southern Vietnam, Western Indonesia including northern Kalimantan and Papau New Guinea (Map created using ﻿QGIS ver.3.22)

Results of year-to-year changes in FC for COVID-2020 versus 2019 for SA/SEA countries are shown in Table 2a,b. Except for Afghanistan, Sri Lanka, and Bangladesh, which had FC increases of 152%, 4.85%, and 0.81%, respectively, during COVID-2020 compared to 2019, all other countries had a decline in FC, with the biggest decline in Nepal (− 73%), Bhutan (− 20%), India (− 11.48%), Pakistan (− 3.38%) (Table 2a). Amongst the Southeast Asian countries, Cambodia (11.1%), Myanmar (8.5%), and Laos (2.78%) had an increase in fires during COVID-2020 compared to 2019. In contrast, there was a decline in Indonesia (− 79%), Malaysia (− 53%), Timor Leste (− 43%), etc. (Table 2b). Spatial variations in percent FC increase or decrease during 2020 versus 2019 for the 0.5° gridded data are shown in Fig. 4. Results suggest the highest percent increase in fires in Afghanistan, Pakistan, and northern Himalayan mountain ranges extending to Nepal with a relatively smaller increase in Eastern Ghats of India, in South Asia. Whereas, in Southeast Asia, Northeast Myanmar, Southern Laos, Southeast Vietnam, Aceh, North-Western Sumatera, northern Kalimantan, Papua-New Guinea in Indonesia showed relatively higher fires during COVID-2020 versus 2019.

**Table 2:** a, b. Percent increase/decrease in VIIRS (375 m) derived fire counts in South/Southeast Asian countries during 2019 versus COVID year 2020.

Countries	2020 COVID year FC	2019 FC	Percent Increase/decrease in FC
**a**			
Afghanistan	2164	856	152.80
Bangladesh	11,054	10,965	0.81
India	470,667	531,727	− 11.48
Pakistan	50,865	52,647	− 3.38
Nepal	8409	31,299	− 73.13
Bhutan	478	605	− 20.99
Sri Lanka	7534	7185	4.85
**b**			
Brunei	183	240	− 23.75
Cambodia	200,712	180,640	11.11
Timor Leste	4217	7421	− 43.17
Indonesia	87,853	427,265	− 79.43
Laos	177,161	172,356	2.78
Malaysia	9333	19,973	− 53.27
Myanmar	409,866	377,443	8.59
Philippines	37,398	33,085	− 11.53
Thailand	219,482	234,860	− 6.54
Vietnam	70,178	72,266	− 2.88

**Figure 5:**
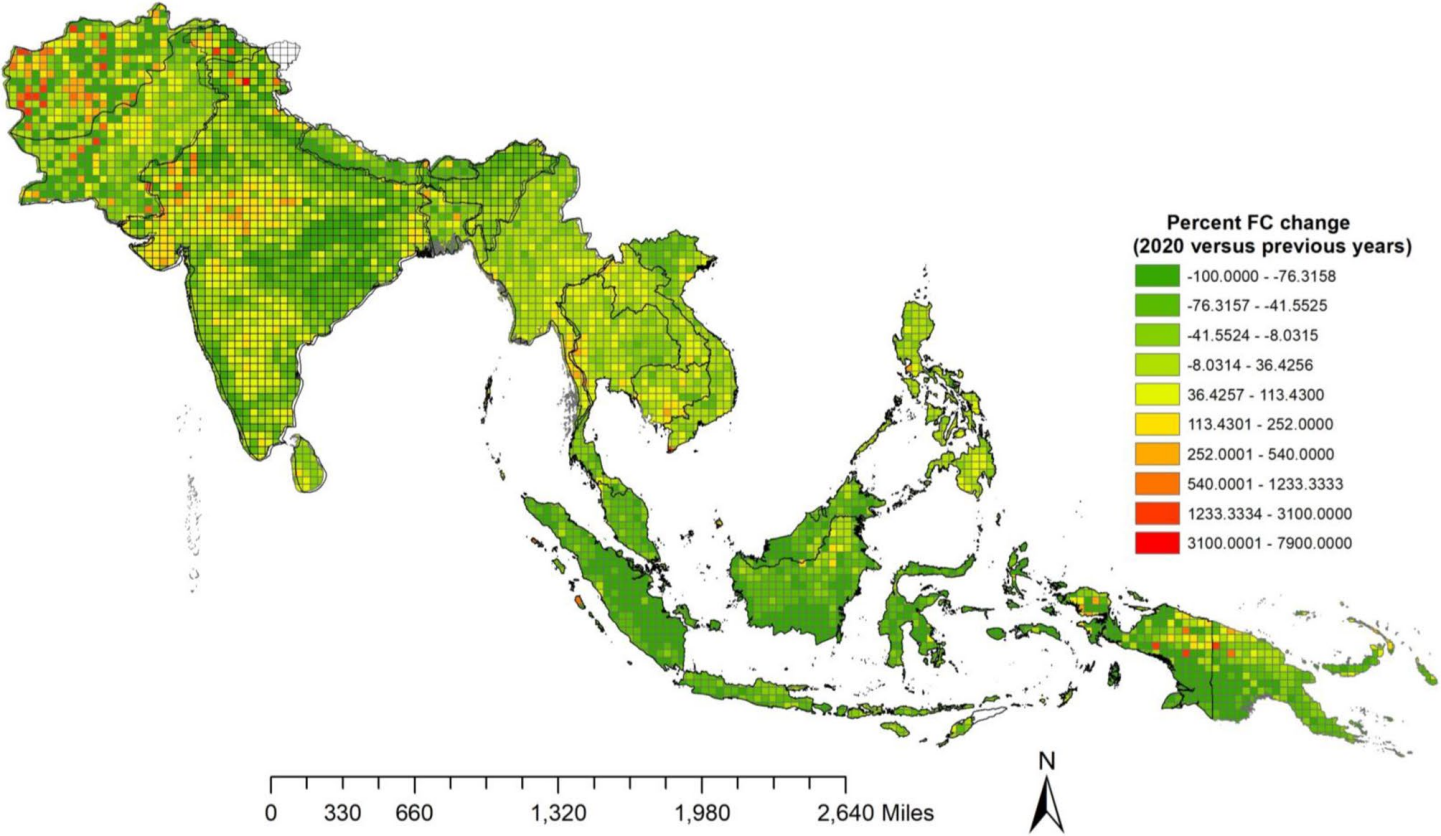
Percent FC change during 2020 versus previous years (2012–2020) for South/Southeast Asia. VIIRS I-band (375 m) fire counts were spatially gridded at 30 min intervals (0.5 × 0.5degree cells) for the regional scale analysis. A clear percent increase in fires can be seen in Afghanistan, Western Pakistan, northern Himalayan region, Central India extending towards south, southern Laos, western Cambodia, Philippines, northern Kalimantan and Papau New Guinea, Indonesia (Map created using ﻿QGIS ver.3.22).

Percent increase or decrease in FC for COVID-2020 year versus pre-pandemic years (2012–2019) for SA/SEA countries are shown in Table 3a,b. Results suggest that except for Afghanistan and Bangladesh, which had an increase in FC of 102.5% and 8.05% during COVID-2020 compared to previous years (2012–2019), the rest of the countries had a decline in FC, with the biggest decline in Nepal (− 70%), Bhutan (− 57%), etc. In the case of SEA countries, an increase in FC during 2020 as compared to the mean FC in previous years (2012–2019) was found for Laos (30.2%), Thailand (21.1%), Myanmar (18.72%), Philippines (13.03%), and Cambodia (7.14), whereas most declines in FC were found for Indonesia (− 74%), Malaysia (− 54.1%), etc. Spatial variations in percent FC increase or decrease during 2020 versus previous years (2012–2019) for the 0.5° gridded data are shown in Fig. 5. An increase in fires can be seen in central and north Western Afghanistan, Central and Western Pakistan, Western and Central India stretching south, northern West Bengal, and Southern Sri Lank in South Asia. In Southeast Asia, Western Cambodia and Southern Myanmar, Southern Laos, Northern Vietnam, both north and the southern Philippines, northern Kalimantan, and Papua New Guinea of Indonesia showed relatively more fires during 2020 than in previous years (2012–2020). Overall, South Asia's mean decrease in fire counts during COVID-2020 compared to 2019 was − 13.23%, whereas 2019 versus earlier years (2012–2018) was only − 6%. Similarly, for the Southeast Asian countries, the mean decline in fire counts during 2020 compared to 2019 was − 25.0%, whereas 2019 versus earlier years (2012–2018) was only − 16%. The new knowledge about the presence of fires consistently (2012–2020) in various regions can help local governmental authorities to intensify their remedial measures and design future strategies for more effective fire control. For example, fire pre-suppression decision requirements are aimed at allocating resources such as firefighting funds, personnel, and equipment. In such a context, the hotspot areas of fires identified in this study can help in fire management and control.

**Table 3:** a, b. Percent increase/decrease in VIIRS (375 m) derived fire counts in South/Southeast Asian countries during 2020 COVID year versus pre-COVID years (2012–2019).

Countries	2020 COVID year FC	Mean Pre − COVID years FC(2012 − 2019)	Percent Increase/decrease in FC
**a**			
Afghanistan	2164	1071	102.05
Bangladesh	11,054	10,229.625	8.05
India	470,667	566,274	− 16.88
Pakistan	50,865	56,270.625	− 9.60
Nepal	8409	28,888.625	− 70.89
Bhutan	478	1137.125	− 57.96
Sri Lanka	7534	8202.25	− 8.14
**b**			
Brunei	183	212	− 13.88
Cambodia	200,712	187,333	7.14
Timor Leste	4217	5833	− 27.70
Indonesia	87,853	345,365	− 74.56
Laos	177,161	135,996	30.26
Malaysia	9333	20,377	− 54.19
Myanmar	409,866	345,234	18.72
Philippines	32,807	33,085	0.84
Thailand	219,482	181,174	21.14
Vietnam	70,178	78,736	− 10.86

**Figure 6:**
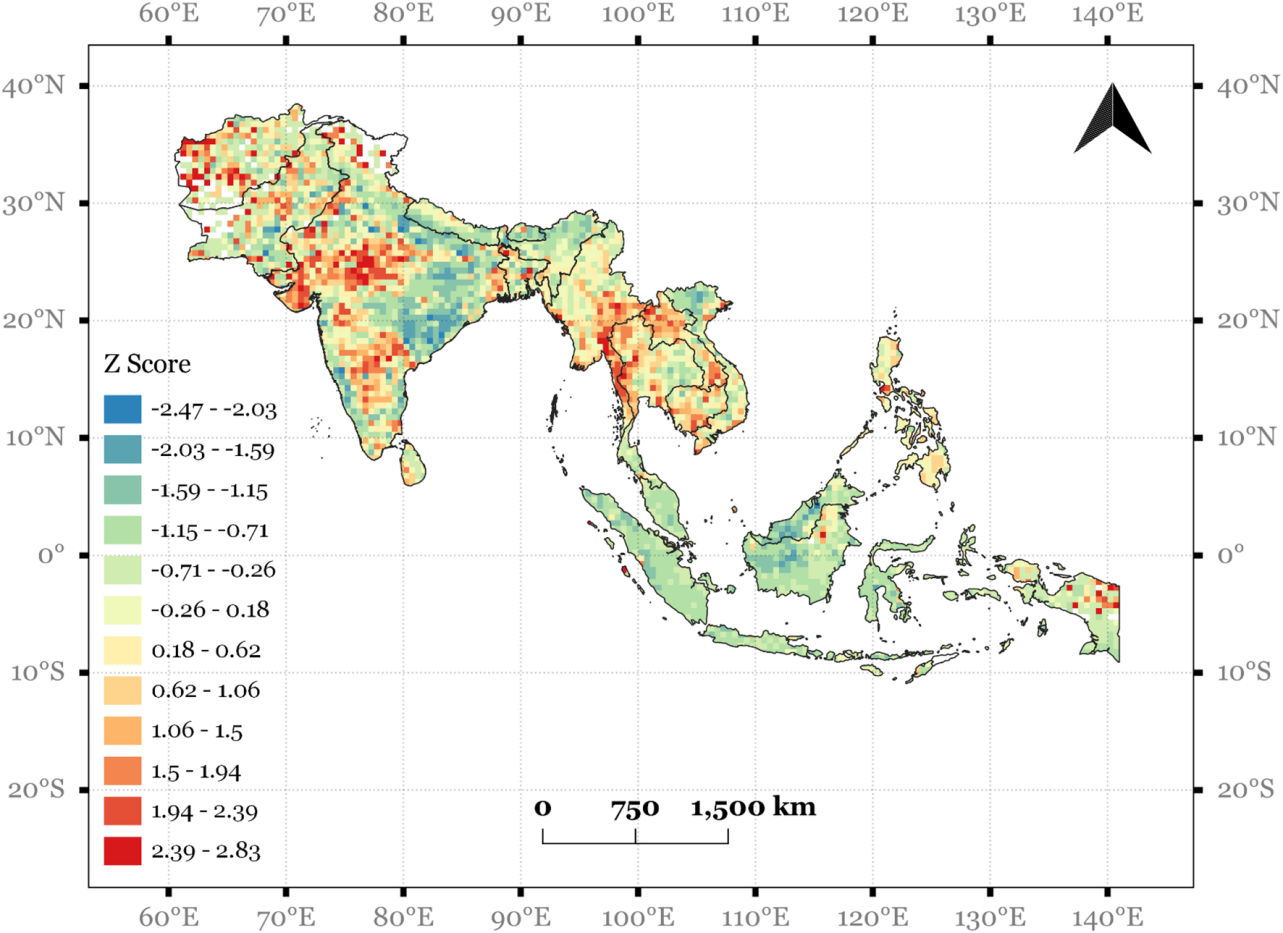
Z-score map of fire counts (*FC*) for 2020 pandemic year derived from VIIRS fire data from 2012–2020. The negative scores indicate places where there was reduced FC and positive values indicate increased *FC* (Map created using QGIS ver.3.22).

**Figure 7:**
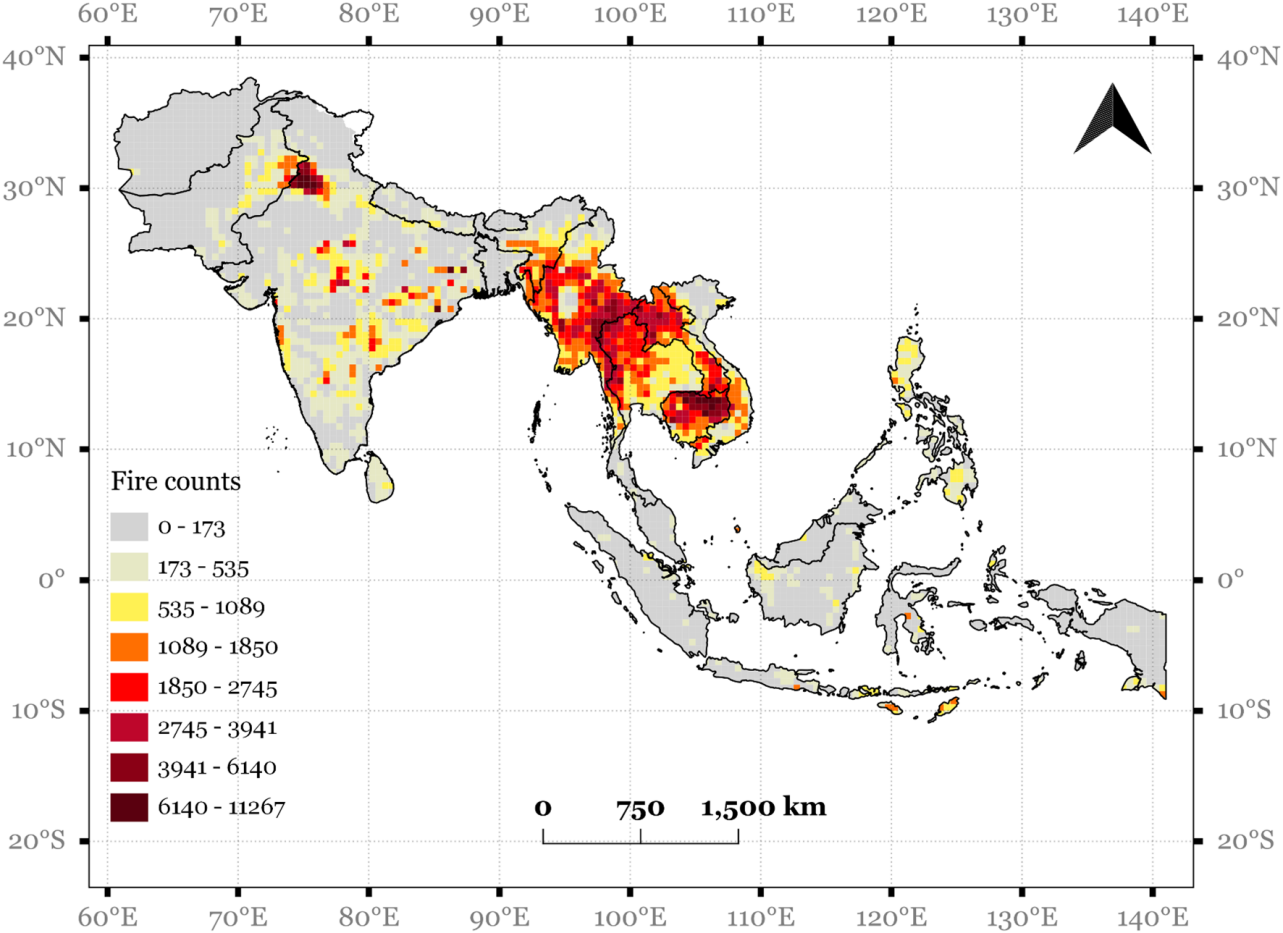
Fire counts (FC) for 2020 pandemic year derived from VIIRS fire data (Map created using QGIS ver.3.22).

Results from the Z-score analysis suggested significant variations at a country level and spatial level (30-min grid cells) (Fig. 6). The original data of FC for 2020 are also shown in Fig. 7. For example, the long-term (2012–2020) data analysis in SA suggested a decline in FC for the 2020 pandemic year for Bhutan (Z =  − 1.02), India (− 1.04), Nepal (− 1.06), Pakistan (− 0.58), Sri Lanka (− 0.24) and increase for Afghanistan (+ 2.30) and Bangladesh (0.30). In SEA, East Timor (− 0.50), Indonesia (− 0.89), Malaysia (− 1.07), Singapore (− 1.2), and Vietnam (− 0.40) had a decline in fires, and Cambodia (*Z* = 0.44), Laos (1.37), Myanmar (1.07), Thailand (0.77) and Philippines (0.07) had an increase in fires. Compared to country-level, several patches in S/SEA showed a decrease in fires for 2020 pandemic year compared to long term 2012–2020 pre-pandemic record, with Z scores greater or less than two denoting statistical significance. Spatially, the decrease in fires with Z-score can be seen in the Eastern and Western parts of India, Northeast India, Northern Myanmar, southern Laos, northern Cambodia, and most parts of Indonesia. On the other hand, the increase in FC can be seen in Central and Southern India, southern Myanmar, northern Thailand, and Southern Cambodia.

### Fire and climate variations

To address whether climate parameters influence the fires, we also analyzed the fire data relationships with the mean monthly temperature (deg.C) and precipitation (mm) for South/Southeast Asia countries from 2012–2020 Figs. 8a,b,c,d,e,f,g, 9a,b,c,d,e,f,g and 10a,b,c,d,e,f,g,h,i,j, 11a,b,c,d,e,f,g,h,i,j respectively. In most South/Southeast Asian countries, fire-temperature and fire-precipitation correlations were poor. For example, only Afghanistan showed a relatively higher positive coefficient of determination (*R*^*2*^ = 0.121) in South Asia, and the others showed poor correlations. Although fire-precipitation plots had a negative slope in South Asia countries, the correlations were poor. In the case of Southeast Asian countries, too, fire-temperature correlations were poor. Relatively, fire-precipitation relationships showed higher negative correlations than temperature. For example, fire-precipitation coefficient of determination (*R*^*2*^) in Indonesia was 0.22, Thailand (0.19), Vietnam (0.18), Myanmar (0.14), Malaysia (0.11), Cambodia (0.10). For the rest of the countries, the correlations were poor. In addition, we also analyzed the temperature (in °C) variations during the peak biomass burning months to infer anomalies at a country scale for 2019 and 2020. Temperature data for different countries are given in the supplementary materials. In South Asian countries, Afghanistan showed a significant increase in fires during 2020 with a corresponding increase in temperature (~ 0.55 °C during 2020), whereas the rest of the countries had a decrease in fires with a corresponding decrease in temperature (< 1.0 °C during 2020). In Southeast Asian countries, except Cambodia and the Philippines, which showed increased fires with increasing temperatures (0.29 and 0.41 °C, respectively) from 2019 to 2020, the rest of the other countries didn't show such a relationship. However, the long-term (2012–2020) fire-precipitation correlations were not strong in these countries. Also, Laos, Myanmar, and Thailand had an increase in fires but a decrease in temperature.

**Figure 8:**
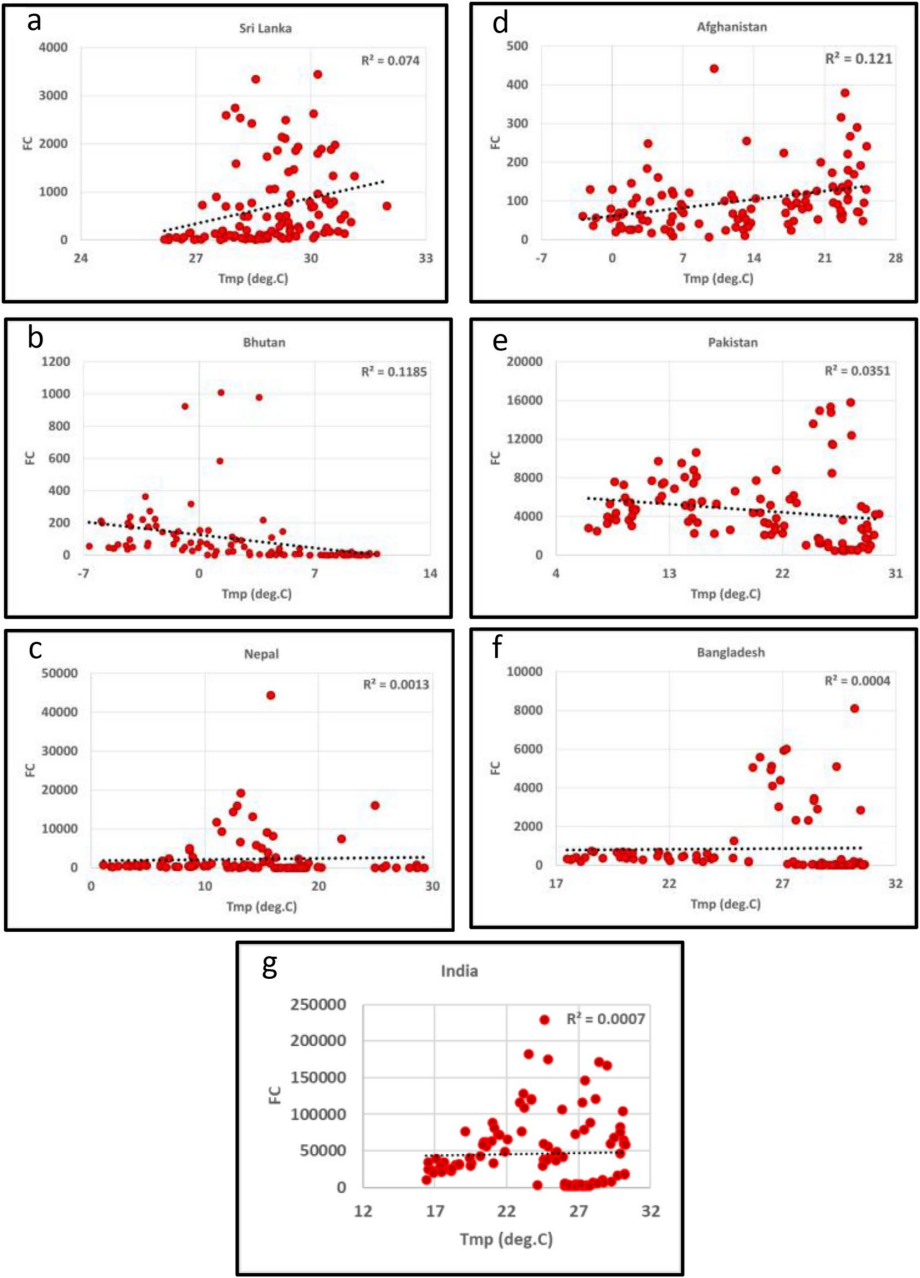
(**a**)–(**g**) Fire Counts (FC)-Temperature (Tmp) relationships in South Asian countries. Most of the correlations were poor.

**Figure 9:**
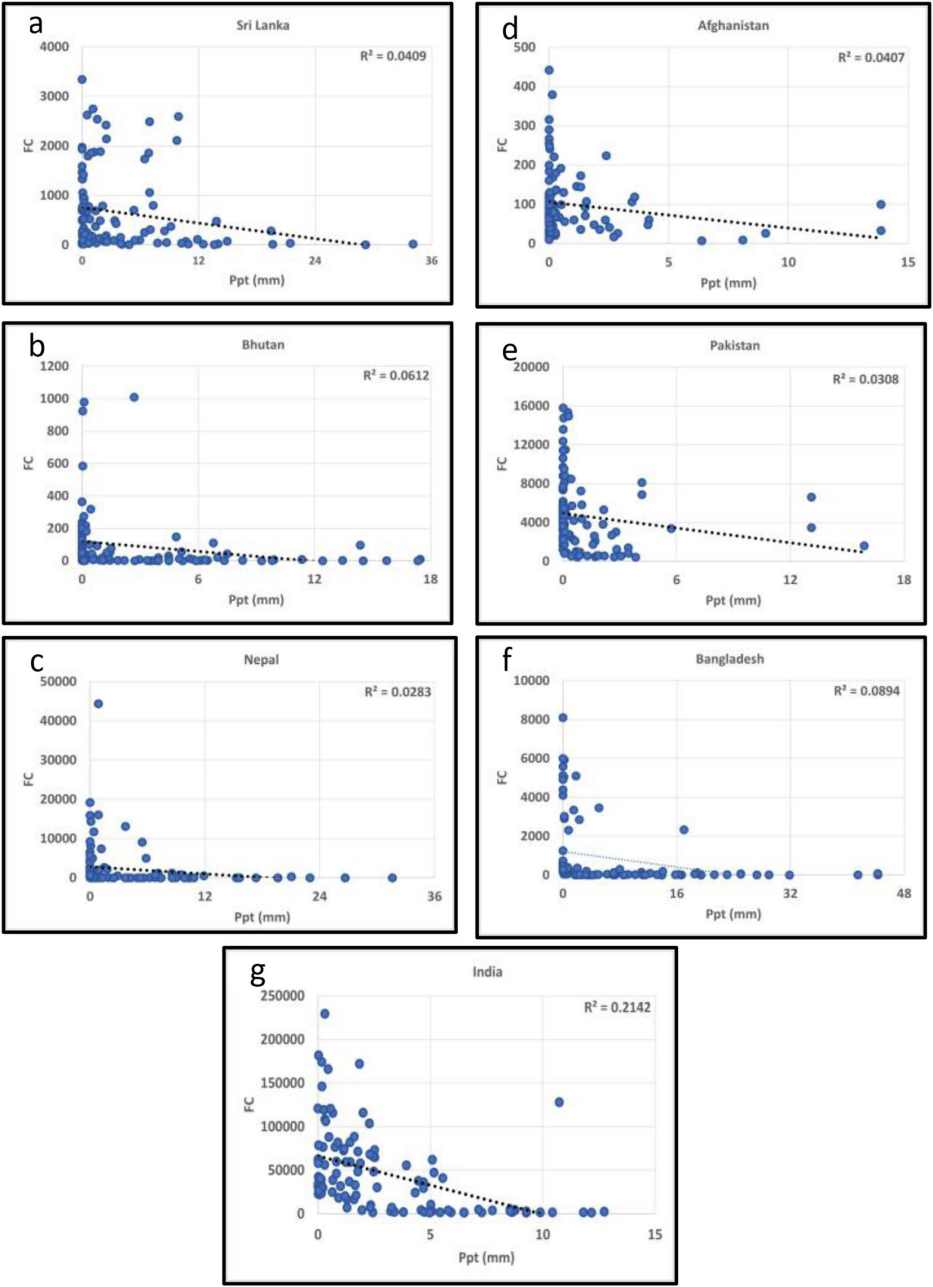
(**a**)–(**g**) Fire Counts (FC)-Precipitation (Ppt) relationships in South Asian countries. Most of the correlations were poor.

**Figure 10:**
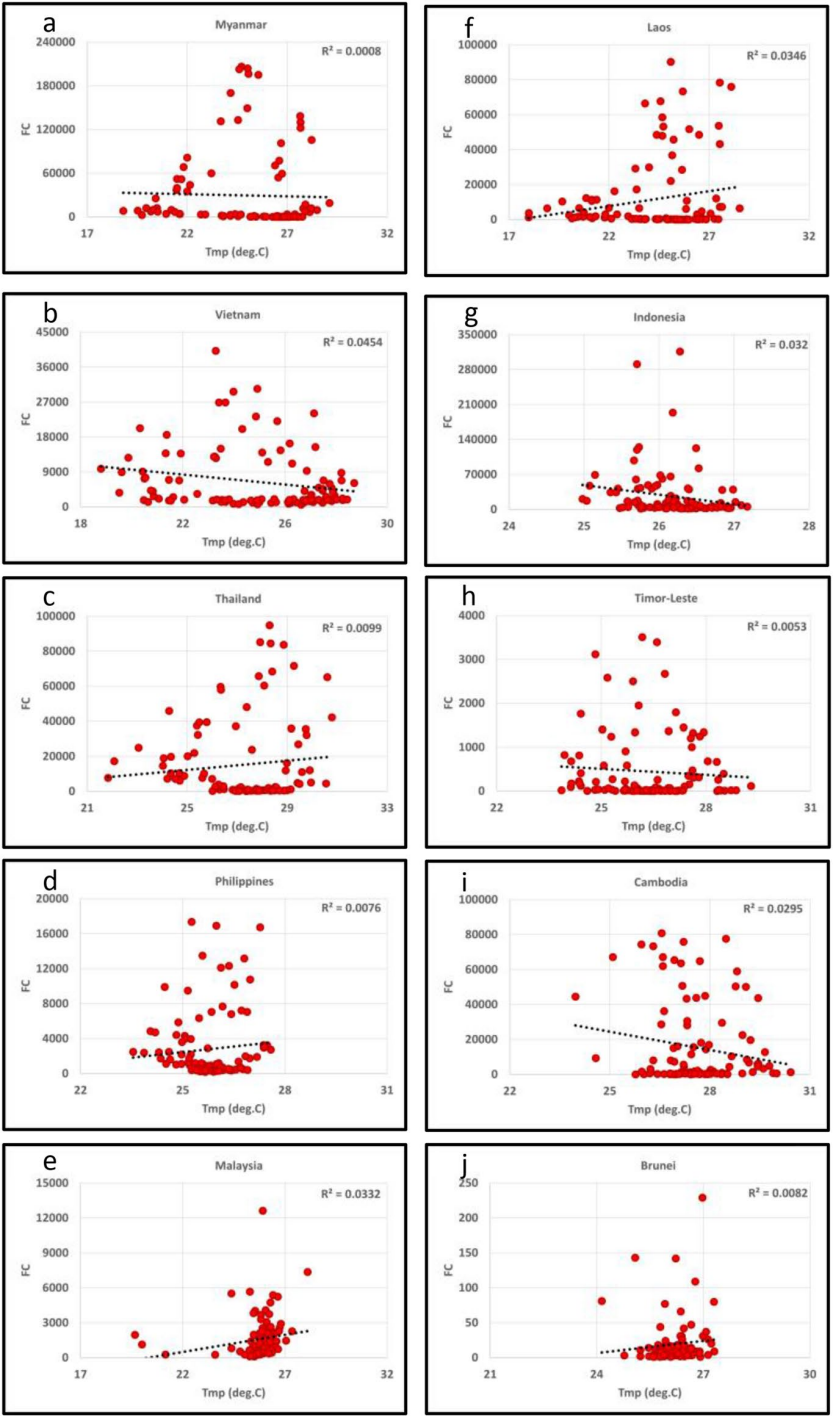
(**a**)–(**j**) Fire Counts (FC)-Temperature (Tmp) relationships in Southeast Asian countries. Most of the correlations were poor.

**Figure 11:**
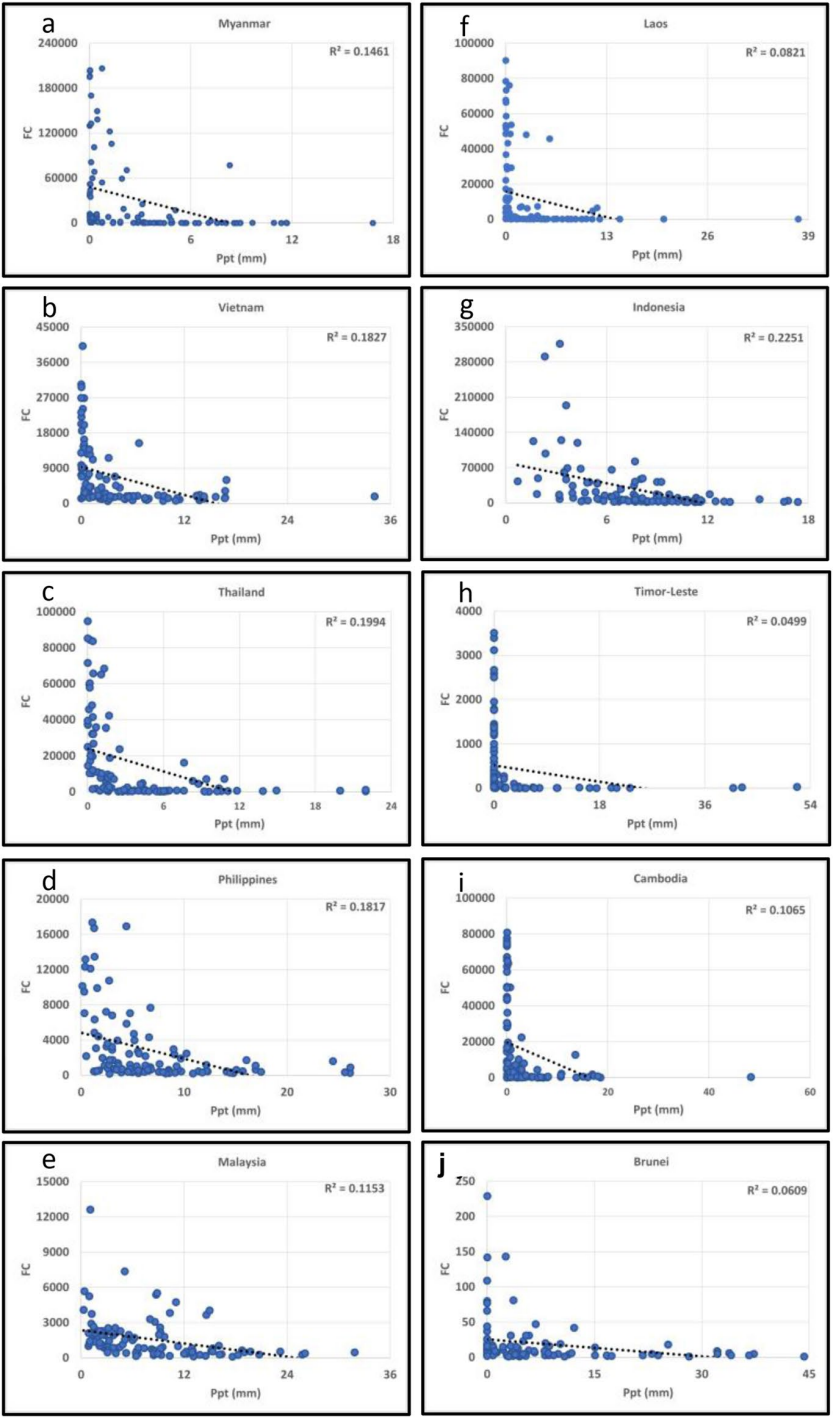
(**a**)–(**j**) Fire Counts (FC)-Precipitation (Ppt) relationships in Southeast Asian countries. Most of the correlations were poor, however, relatively, FC showed higher coefficient of determination with precipitation than temperature.

### Fires and emissions

**Figure 12:**
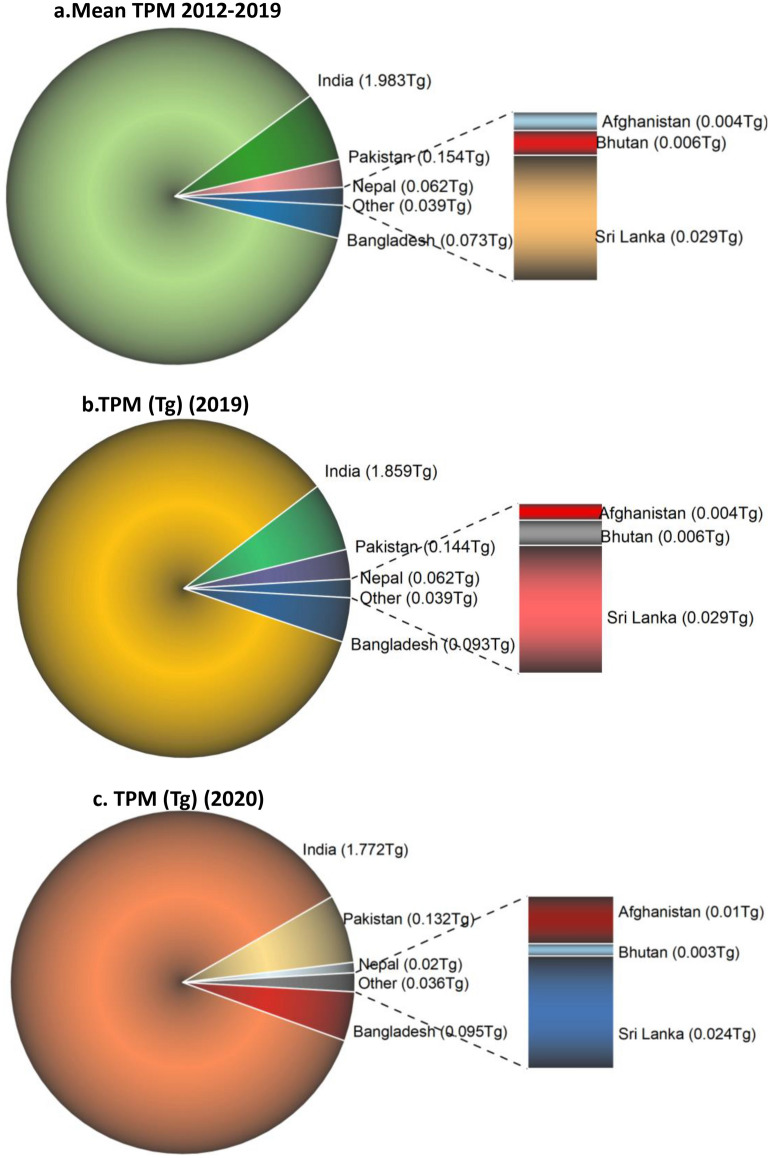
(**a**)–(**c**) Total Particulate Matter (TPM) emissions (Tg) from South Asian countries. (**a**). Mean TPM (Tg) during 2012–2019; (**b**). 2019-non COVID year; (**c**). 2020-COVID year. A reduction in TPM emissions has been observed during COVID-2020 for most of the countries, except Afghanistan and Bangladesh. 2020-COVID year had an overall reduction of ~ 0.26 Tg TPM emissions compared to previous non-COVID years and 0.14 Tg less than 2019 non-COVID year.

Results on the TPM emissions (in Tg) for different SA countries are shown for 2012–2019, 2019, and 2020 in Fig. 12a,b,c. Results suggest a mean of ~ 2.31 Tg TPM for 2012–2019 from the South Asian countries. Of the 2.31Tg, ~ 86% is contributed by India, followed by Pakistan (6.64%), Bangladesh (3.1%), Nepal (2.6%), Sri Lanka (1.2%), and Afghanistan (0.18%). For 2019, the total TPM from South Asian countries is about 2.19Tg, i.e., 0.11Tg less than the mean TPM emissions from previous years. Further, in 2020 the total TPM was about 2.05Tg, i.e., 0.261 less than the previous years and 0.14Tg less than 2019. Overall, compared to TPM emissions in 2012–2019, in terms of percent decline during 2020, Nepal had the highest drop (− 67.2%), followed by Bhutan (− 54.6%), Pakistan (− 13.7%), whereas Afghanistan and Bangladesh had an increase in TPM of 127% and 31% during 2020.

**Figure 13:**
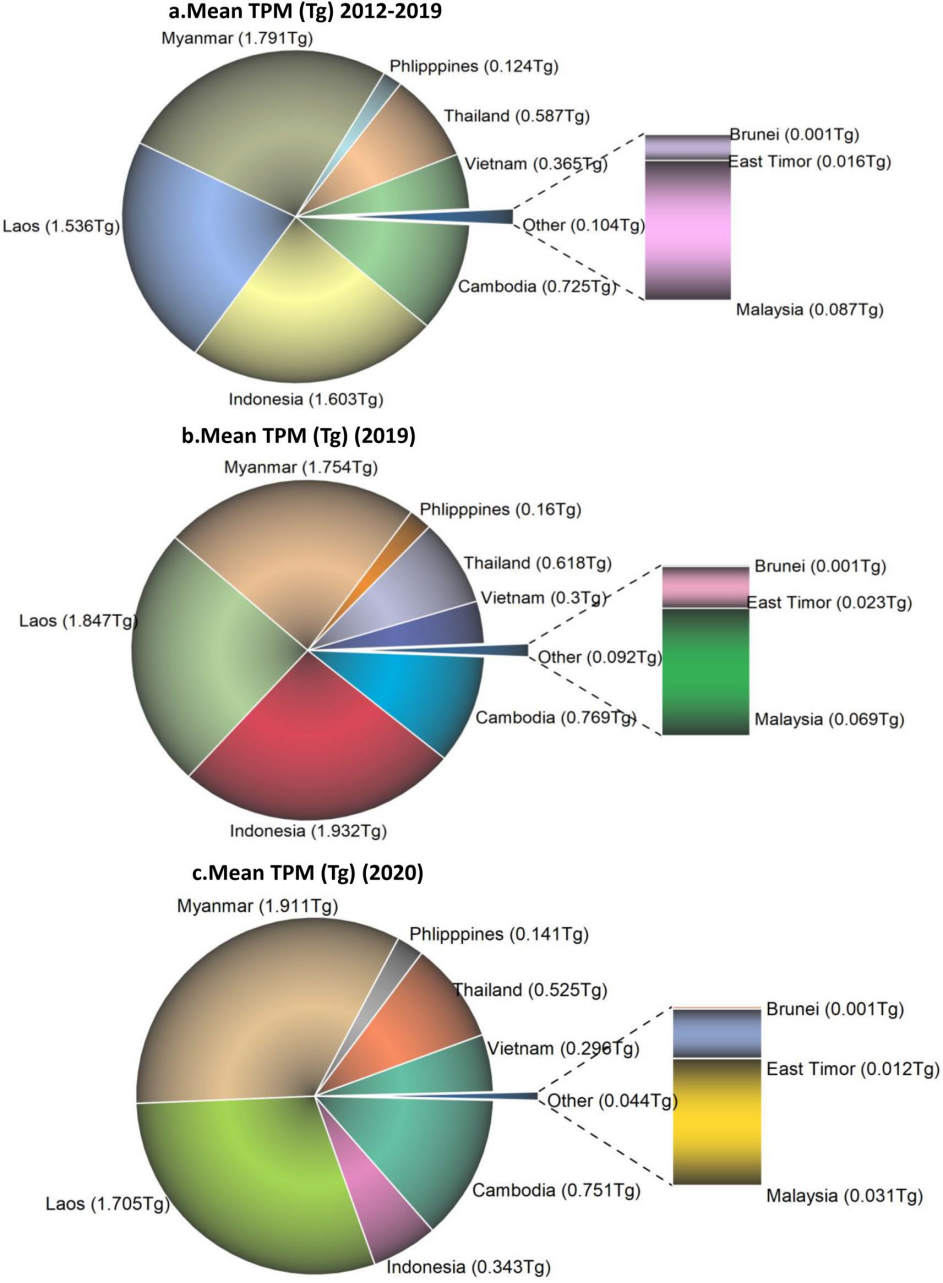
(**a**)–(**c**) Total Particulate Matter (TPM) emissions (Tg) from Southeast Asian countries. (**a**). Mean TPM (Tg) during 2012–2019; (**b**). 2019-non COVID year; (**c**). 2020-COVID year. A reduction in TPM emissions has been observed during COVID-2020 for most of the countries, except Myanmar, Laos, Cambodia and Philippines. 2020-COVID year had an overall reduction of ~ 1.11 Tg TPM emissions compared to previous non-COVID years and 1.75 Tg less than 2019 non-COVID year.

Results on the TPM emissions for different SEA countries are shown for 2012–2019, 2019 and 2020 in Fig. 13,a,b,c. Results show a mean of ~ 6.83 Tg TPM for 2012–2019 from the Southeast Asian countries. Of the total 6.83 Tg, ~ 26% is contributed by Myanmar followed by Indonesia (23.4%), Laos (22.4%), Cambodia (10.61%), Thailand (8.59%), Vietnam (5.34%), Philippines (1.81%), Malaysia (1.27%), Timor Leste (0.23%) and Brunei (0.01%). For 2019, the total TPM from Southeast Asian countries is about 7.47Tg, i.e., 0.64Tg higher than the mean TPM emissions from previous years. Further, for 2020, the total TPM was about 5.716Tg, i.e., 1.11Tg less than the previous non-COVID years and 1.75Tg less than 2019. Overall, comparing TPM emissions during 2012–2019 to 2020, Indonesia had the highest decline (− 78.59%), followed by Malaysia (− 64.0%), Timor Leste (− 24.9%), etc., whereas the Philippines had an increase of 13.9% in TPM emissions followed by Myanmar (6.70%), Laos (11.0%), Cambodia (3.60%). Further, a comparison of TPM emissions suggested relatively higher emissions from Southeast Asian countries (5.71 Tg) than South Asian countries (2.05Tg) during 2020.

## Discussion

Most of the fires in SA/SEA are attributed to anthropogenic factors^10–12^. The poor correlations between the fires and climate variables confirm these relationships. Due to the COVID pandemic, several countries implemented stringent measures on people's movement from one region to another; thus, there is a good chance there has been less pressure on the natural ecosystems^60,61^. However, given the relatively short period, such impacts are not clearly documented in the literature. Only one study of which the authors are aware compared fire data during COVID-2020 versus previous years in the SA/SEA region. The study in Nepal found that during the pandemic, there was a 4.54% decrease in forest fire incidents and an 11.36% reduction in FRP associated with these events, showing that districts with smaller areas of community-managed forests per capita experienced an 8.11% decrease in the number of forest fire incidents^60^. Our results indicate that most countries in SA/SEA had reduced fires during 2020 compared to 2019. However, a more thorough analysis comparing the relatively long-term fire datasets (2012–2019) and inter-annual variability suggested that the decline in fires during 2020 was not statistically significant for most countries. We also analyzed whether temperature variations influenced the FC in different countries. We noted that temperature variations in South Asian countries are relatively more associated with an increase (Afghanistan) or decrease (in other countries) in SA than in SEA countries. It is important to note that we did not analyze other important drivers such as El Nino Southern Oscillation (ENSO) in SEA, which is a limitation. Specific to ENSO in the region and Indonesia, it was shown to be a drought-inducing agent that, combined with human drivers, affects fires spatially and temporally^62–64^. Analysis of ENSO in relation to fires requires separation of land and sea-side domains separately for ENSO impacts, including lagged-correlations. In our study, we aggregated the data for different years and analyzed the correlations with the country-wide temperature and precipitation data. In contrast, the most common indicator for ENSO is sea surface temperature variations. Further, separating climate data versus human-induced fires requires land use spatially per grid cell. Although, analyzing the data for individual years and all such drivers is beyond the scope of the study, we realize the importance of such drivers. Since most of the fires in Asia are anthropogenic in nature, the temporary decline in fires that we observed during 2020 compared to 2019 may be attributed to COVID-related travel restrictions. Specifically, severe restrictions placed on personal travel might have impacted rural areas regarding consumer and business services and visitors to rural areas impacting natural ecosystems^34,35,61^. It may be noted that most of the fires observed in both SA/SEA are related to the agriculture sector, mainly due to clearing agricultural residues after harvest^10^. Due to COVID restrictions lockdowns (see Table 1, supplementary materials), the agriculture sector was affected, more specifically due to labor shortage for agriculture work, including planting, harvesting, transportation, marketing, and processing^65,66^. For example, a recent study highlighted that in India, around 44% of the income in rural communities is from wage labor, compared to only 23% from crop cultivation and livestock and that most of the labor is from migrant workers from cities^35,67^. Due to the COVID lockdown, the labor shortage might have reduced post-harvest residue management practices, including crop residue fires in specific regions of the country. Also, forest-related tourism and visitor attractions to natural areas might have slowed down due to COVID-19 travel restrictions in several countries, which might have reduced accidental fires. More thorough social surveys are needed to confirm these inferences. Unfortunately, the socioeconomic and policy data, particularly the temporal ones coinciding with the fires, were unavailable to confirm these inferences.

The use of 375-m VIIRS fire data, including the associated FRP information to characterize fire characteristics in the current study, is appropriate due to the superior capabilities of VIIRS data in fire detection compared to the MODIS datasets. Details on the VIIRS 375-m fire detection algorithm's theoretical performance are given in^31^. The VIIRS algorithm theoretical minimum detectable limit for night fires is equivalent to ~ 5 m^2^ and ~ 1000 K resulting from homogeneous background conditions inferred from the experimental fires. Also^31^, noted that the day and nighttime contrast in the background helps in characterizing fire diurnal variations. Specific to Asia in Borneo, the false alarm rate was almost 0%, and the daytime low confidence fire pixels account for approximately 11% of all daytime detections produced globally. A recent study^68^ on FRP variations comparing MODIS and VIIRS suggests that while MODIS can detect fires with FRP of ~ 4.3 MW (per pixel) at nadir and > 31.7 MW at the scan edge, VIIRS can sense fires with FRP of ~ 1.3 MW (per pixel) and > 2.9 MW, respectively, and that VIIRS I band (375 m) can detect fires that are approximately 3–11 times less intense. These unique qualities of the VIIRS fire product help characterize cropland fires most common in South/Southeast Asia, which have relatively lower FRP than forest fires.

Using the VIIRS FRP, we quantified TPM emissions and observed an overall decline in emissions in several SA/SEA countries during the 2020-COVID year. In this study, rather than using the traditional approach of quantifying fire related emissions using ground-based biomass data, satellite derived burned areas, and emission factors, we preferred the FRP based approach as it is more direct, and ground-based information is not required. In particular, during this COVID pandemic, obtaining such ground-based data was not feasible. Thus, the TPM emission coefficients that are based on regionally collocated satellite measurements of both FRP and aerosol optical thickness (AOT) are quite helpful in quantifying the fire-related TPM emissions for different years, including COVID-2020. The approach helped us highlight total TPM emissions (in Tg) and regional and country scale variations. However, converting FRP to FRE to capture the diurnal fire cycle using the geostationary data could not be attempted in the study as such data with high temporal and spatial repeat capability for all S/SEA countries over the time domain were not available. Thus, we used only VIIRS fire observations and used a three-hour fire cycle assumption considering dominant agriculture fires with a mix of other vegetation fires in the region which might have resulted in underestimation on the overall TPM emissions. Despite these limitations, our study highlights the impact of fire footprint on the environment in SA/SEA. Results highlight a temporary reduction in TPM emissions during the 2020 pandemic year from fires compared to the 2019 non-pandemic year. However, as the economy reopened, fire-related emissions will likely rebound without the lockdown measures. In addition, more socioeconomic data such as population demographics, migration, and policy measures are needed to understand regional fire variations useful for fire management and mitigation.

## Conclusions

We investigated variations in vegetation fires in South/Southeast Asia during the COVID-19 pandemic (2020) compared to recent pre-pandemic years (2012–2019) and the year before the pandemic. Our analysis demonstrates that there has been a decline in fires in most countries during 2020 compared to 2019. However, a more thorough analysis comparing the relatively long-term fire datasets (2012–2019) suggested that the decline in fires during 2020 was not statistically significant for most countries. We also analyzed active fire data in relation to temperature and precipitation variations. Precipitation had relatively more negative correlations in Southeast Asian countries than South Asia. Overall the satellite datasets were quite helpful in capturing fire variations before and after the COVID pandemic. We also provided fire related total particulate matter (TPM) emissions in the study. The total TPM from fires in South Asian countries was 2.19 Tg which decreased to 2.05 Tg, i.e., a decline of 0.14 Tg. Similarly, in Southeast Asian countries, the total TPM from Southeast Asian countries is about 7.47 Tg which decreased to 5.716 Tg in 2020, i.e., 1.75 Tg less than in 2019. Addressing inter-annual variations in fires, including the recent decline in 2020, requires additional data such as demographics, migration, and land use policies useful for fire management and mitigation in the region. Nevertheless, irrespective of the drivers, the overall reduction in fires and related emissions in 2020 compared to 2019 had a positive environmental impact, with less pollution in some South/Southeast Asian countries.

## Supplementary Information

Supplementary Information.

## Data Availability

The datasets generated during and/or analyzed during the current study are mostly presented in the supplementary material. Further inquiries can be directed to the corresponding author.

## References

[1] Baker PJ, Bunyavejchewin S (2009). Fire behavior and fire effects across the forest landscape of continental Southeast Asia. Tropical Fire Ecology.

[2] Sodhi NS (2004). Southeast Asian biodiversity: an impending disaster. Trends Ecol. Evol..

[3] Inoue J, Okuyama C, Takemura K (2018). Long-term fire activity under the East Asian monsoon responding to spring insolation, vegetation type, global climate, and human impact inferred from charcoal records in Lake Biwa sediments in central Japan. Quat. Sci. Rev..

[4] Goldammer JG (2012). Fire in the Tropical Biota: Ecosystem Processes and Global Challenges.

[5] Taylor AH (2010). Fire disturbance and forest structure in an old-growth Pinus ponderosa forest, southern Cascades. USA. J. Veg. Sci..

[6] Bowman DMJS (2009). Fire in the Earth system. Science.

[7] Adams MA (2013). Mega-fires, tipping points and ecosystem services: Managing forests and woodlands in an uncertain future. For. Ecol. Manage..

[8] Crutzen PJ, Andreae MO (1990). Biomass burning in the tropics: Impact on atmospheric chemistry and biogeochemical cycles. Science.

[9] Prasad VK, Badarinath KVS, Eaturu A (2008). Biophysical and anthropogenic controls of forest fires in the Deccan Plateau. India. J. Environ. Manage..

[10] Vadrevu KP (2019). Trends in vegetation fires in south and Southeast Asian Countries. Sci. Rep..

[11] Mukul SA, Byg A (2020). What determines indigenous Chepang farmers' Swidden land-use decisions in the central hill districts of Nepal?. Sustainability..

[12] Prasad VK, Kant Y, Gupta PK, Sharma C, Mitra AA, Badarinath KVS (2001). Biomass and combustion characteristics of secondary mixed deciduous forests in Eastern Ghats of India. Atmos. Environ..

[13] Toky OP, Ramakrishnan PS (1983). Secondary succession following slash and burn agriculture in North-Eastern India: I. Biomass, litterfall and productivity. J. Ecol..

[14] Borggaard OK, Gafur A, Petersen L (2003). Sustainability appraisal of shifting cultivation in the Chittagong hill tracts of Bangladesh. AMBIO J. Hum. Environ..

[15] Biswas S (2015). Factors controlling vegetation fires in protected and non-protected areas of Myanmar. PLoS ONE.

[16] de Neergaard A, Magid J, Mertz O (2008). Soil erosion from shifting cultivation and other smallholder land use in Sarawak, Malaysia. Agr. Ecosyst. Environ..

[17] Gabriel AG, De Vera M, Antonio MAB (2020). Roles of indigenous women in forest conservation: A comparative analysis of two indigenous communities in the Philippines. Cogent Soc. Sci..

[18] Ketterings QM, van Noordwijk M, Bigham JM (2002). Soil phosphorus availability after slash-and-burn fires of different intensities in rubber agroforests in Sumatra, Indonesia. Agr. Ecosyst. Environ..

[19] Grandstaff, T. B. 1980. Shifting cultivation in northern Thailand. Possibilities for development. No.3. Cabdirect.org.

[20] Scheidel, A. and Work, C., 2016. Large-scale forest plantations for climate change mitigation? New frontiers of deforestation and land grabbing in Cambodia. Global Governance/Politics, Climate Justice and Agrarian/Social Justice: Linkages and Challenges. 11.

[21] Inoue Y, Vadrevu KP, Ohara T, Justice C (2018). Ecosystem carbon stock, atmosphere, and food security in slash-and-burn land use: A geospatial study in a mountainous region of Laos. Land-Atmospheric Research Applications in South and Southeast Asia.

[22] Lasko K (2017). Satellites may underestimate rice residue and associated burning emissions in Vietnam. Environ. Res. Lett..

[23] Dhandapani S, Evers S (2020). Oil palm' slash-and-burn'practice increases post-fire greenhouse gas emissions and nutrient concentrations in burnt regions of an agricultural tropical peatland. Sci. Total Environ..

[24] Roengtam S, Agustiyara A (2022). Collaborative governance for forest land use policy implementation and development. Cogent Soc. Sci..

[25] Van der Werf GR, Dempewolf J, Trigg SN, Randerson JT, Kasibhatla PS, Giglio L, Murdiyarso D, Peters W, Morton DC, Collatz GJ, Dolman AJ (2008). Climate regulation of fire emissions and deforestation in equatorial Asia. Proc. Natl. Acad. Sci..

[26] Radojevic M (2003). Chemistry of forest fires and regional haze with emphasis on Southeast Asia. Pure Appl. Geophys..

[27] Dwyer E (2000). Global spatial and temporal distribution of vegetation fire as determined from satellite observations. Int. J. Remote Sens..

[28] Goldammer J, Statheropoulos M, Andreae MO (2008). Impacts of vegetation fire emissions on the environment, human health and security: A global perspective. Developments in environmental science.

[29] Matson M, Holben B (1987). Satellite detection of tropical burning in Brazil. Int. J. Remote Sens..

[30] Giglio L, Schroeder W, Justice CO (2016). The collection 6 MODIS active fire detection algorithm and fire products. Remote Sens. Environ..

[31] Schroeder W (2014). The New VIIRS 375 m active fire detection data product: Algorithm description and initial assessment. Remote Sens. Environ..

[32] Huang C (2020). Clinical features of patients infected with 2019 novel coronavirus in Wuhan. China. Lancet.

[33] World Health Organization. COVID-19 Weekly Epidemiological Update Global overview. https://apps.who.int/iris/bitstream/handle/10665/339858/nCoV-weekly-sitrep23Feb21-eng.pdf?sequence=1 (2021).

[34] Anzai A (2020). Assessing the impact of reduced travel on exportation dynamics of novel coronavirus infection (COVID-19). J. Clin. Med..

[35] Phillipson J (2020). The COVID-19 pandemic and its implications for rural economies. Sustainability.

[36] Singh V (2020). Diurnal and temporal changes in air pollution during COVID-19 strict lockdown over different regions of India. Environ. Pollut..

[37] Vadrevu KP (2020). Spatial and temporal variations of air pollution over 41 cities of India during the COVID-19 lockdown period. Sci. Rep..

[38] Kanniah KD (2020). COVID-19's impact on the atmospheric environment in the Southeast Asia region. Sci. Total Environ..

[39] Ghahremanloo M (2021). Impact of the COVID-19 outbreak on air pollution levels in East Asia. Sci. Total Environ..

[40] Kaufman YJ (1998). Potential global fire monitoring from EOS-MODIS. J. Geophys. Res. Atmos..

[41] Giglio L (2003). An enhanced contextual fire detection algorithm for MODIS. Remote Sens. Environ..

[42] Giglio L (2009). An active-fire based burned area mapping algorithm for the MODIS sensor. Remote Sens. Environ..

[43] Giglio L (2018). The collection 6 MODIS burned area mapping algorithm and product. Remote Sens. Environ..

[44] Wooster MJ, Zhukov B, Oertel D (2003). Fire radiative energy for quantitative study of biomass burning: Derivation from the BIRD experimental satellite and comparison to MODIS fire products. Remote Sens. Environ..

[45] Wooster MJ (2005). Retrieval of biomass combustion rates and totals from fire radiative power observations: FRP derivation and calibration relationships between biomass consumption and fire radiative energy release. J. Geophys. Res..

[46] Ichoku C (2008). Global characterization of biomass-burning patterns using satellite measurements of fire radiative energy. Remote Sens. Environ..

[47] Ichoku C (2005). Quantitative evaluation and intercomparison of morning and afternoon moderate resolution imaging spectroradiometer (MODIS) aerosol measurements from Terra and Aqua. J. Geophys. Res..

[48] Vermote E (2009). An approach to estimate global biomass burning emissions of organic and black carbon from MODIS fire radiative power. J. Geophys. Res..

[49] Wooster MJ (2002). Small-scale experimental testing of fire radiative energy for quantifying mass combusted in natural vegetation fires. Geophys. Res. Lett..

[50] Zhang T (2018). How well does the 'small fire boost' methodology used within the GFED41s fire emissions database represent the timing, location and magnitude of agricultural burning?. Remote Sens..

[51] Roberts, G., Wooster, M. J. & Lagoudakis, E. Annual and diurnal african biomass burning temporal dynamics. Biogeosciences vol. 6 www.biogeosciences.net/6/849/2009/ (2009).

[52] Zhang, X. et al. Near-real-time global biomass burning emissions product from geostationary satellite constellation. *J*. *Geophys*. *Res*. *Atmos*. **117**, n/a-n/a (2012).

[53] Ellicott E (2009). Estimating biomass consumed from fire using MODIS FRE. Geophys. Res. Lett..

[54] Freeborn PH (2008). Relationships between energy release, fuel mass loss, and trace gas and aerosol emissions during laboratory biomass fires. J. Geophys. Res..

[55] Nguyen HM, Wooster MJ (2020). Advances in the estimation of high Spatio-temporal resolution pan-African top-down biomass burning emissions made using geostationary fire radiative power (FRP) and MAIAC aerosol optical depth (AOD) data. Remote Sens. Environ..

[56] Ichoku C, Kaufman YJ (2005). A method to derive smoke emission rates from MODIS fire radiative energy measurements. IEEE Trans. Geosci. Remote Sens..

[57] Ichoku C, Ellison L (2014). Global top-down smoke-aerosol emissions estimation using satellite fire radiative power measurements. Atmos. Chem. Phys..

[58] Mota B, Wooster MJ (2018). A new top-down approach for directly estimating biomass burning emissions and fuel consumption rates and totals from geostationary satellite fire radiative power (FRP). Remote Sens. Environ..

[59] Nguyen HM, Wooster MJ (2020). Advances in the estimation of high Spatio-temporal resolution pan-African top-down biomass burning emissions made using geostationary fire radiative power (FRP) and MAIAC aerosol optical depth (AOD) data. Remote Sensing of Environment.

[60] Paudel J (2021). Short-run environmental effects of COVID-19: Evidence from forest fires. World Dev..

[61] Setiati S, Azwar MK (2020). COVID-19 and Indonesia. Acta Med Indones-Indones J. Intern. Med..

[62] Spessa AC, Field RD, Pappenberger F, Langner A, Englhart S, Weber U, Stockdale T, Siegert F, Kaiser JW, Moore J (2015). Seasonal forecasting of fire over Kalimantan, Indonesia. Nat. Hazard..

[63] Field RD, Van Der Werf GR, Fanin T, Fetzer EJ, Fuller R, Jethva H, Levy R, Livesey NJ, Luo M, Torres O, Worden HM (2016). Indonesian fire activity and smoke pollution in 2015 show persistent nonlinear sensitivity to El Niño-induced drought. Proc. Natl. Acad. Sci..

[64] Nurdiati S, Bukhari F, Julianto MT, Sopaheluwakan A, Aprilia M, Fajar I, Septiawan P, Najib MK (2022). The impact of El Niño southern oscillation and Indian Ocean Dipole on the burned area in Indonesia. Terr. Atmos. Ocean. Sci..

[65] Gregorioa GB, Ancog RC (2020). Assessing the impact of the covid-19 pandemic on agricultural production in Southeast Asia: Toward transformative change in agricultural food systems. Asian J. Agric. Dev..

[66] Kumar P (2021). Multi-level impacts of the COVID-19 lockdown on agricultural systems in India: The case of Uttar Pradesh. Agric. Syst..

[67] Agarwal S, Singh A (2020). Covid-19 and its impact on Indian economy. Int. J. Trade Comm. IIARTC.

[68] Li F, Zhang X, Kondragunta S (2020). Biomass burning in Africa: An investigation of fire radiative power missed by MODIS using the 375 m VIIRS active fire product. Remote Sens..

