# Supplementary material for: Spatial variations in vegetation fires and emissions in South and Southeast Asia during COVID-19 and pre-pandemic
Source: Sci Rep. 2022 Oct 29;12:18233. doi: 10.1038/s41598-022-22834-5 (PMC9617248; doi:10.1038/s41598-022-22834-5)
Supplement: Supplementary file 1 — Supplementary Information. [file 41598_2022_22834_MOESM1_ESM.pptx]

## Slide 1
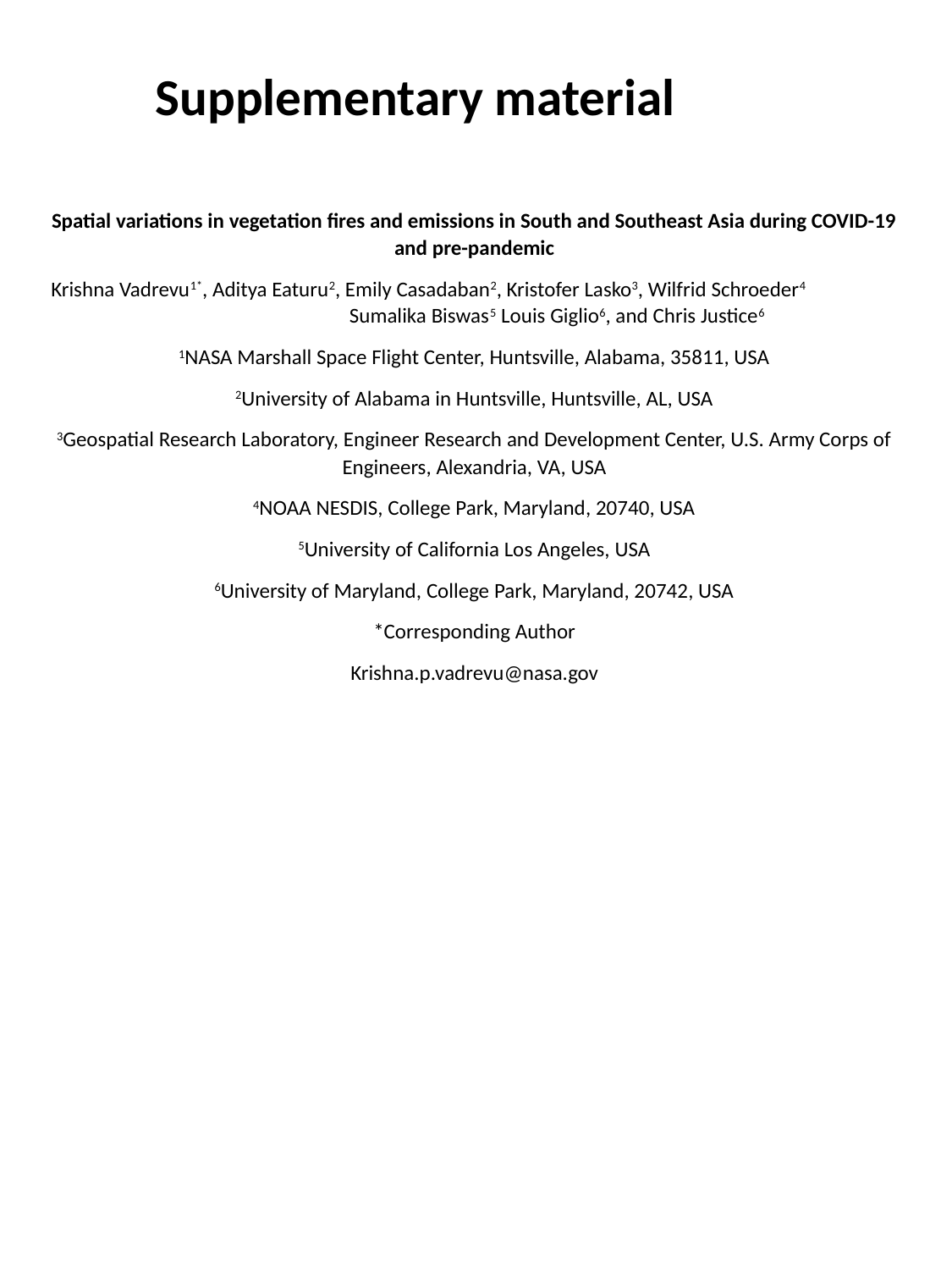

Supplementary material
Spatial variations in vegetation fires and emissions in South and Southeast Asia during COVID-19 and pre-pandemic
Krishna Vadrevu1*, Aditya Eaturu2, Emily Casadaban2, Kristofer Lasko3, Wilfrid Schroeder4 Sumalika Biswas5 Louis Giglio6, and Chris Justice6
1NASA Marshall Space Flight Center, Huntsville, Alabama, 35811, USA
2University of Alabama in Huntsville, Huntsville, AL, USA
3Geospatial Research Laboratory, Engineer Research and Development Center, U.S. Army Corps of Engineers, Alexandria, VA, USA
4NOAA NESDIS, College Park, Maryland, 20740, USA
5University of California Los Angeles, USA
6University of Maryland, College Park, Maryland, 20742, USA
*Corresponding Author
Krishna.p.vadrevu@nasa.gov

## Slide 2
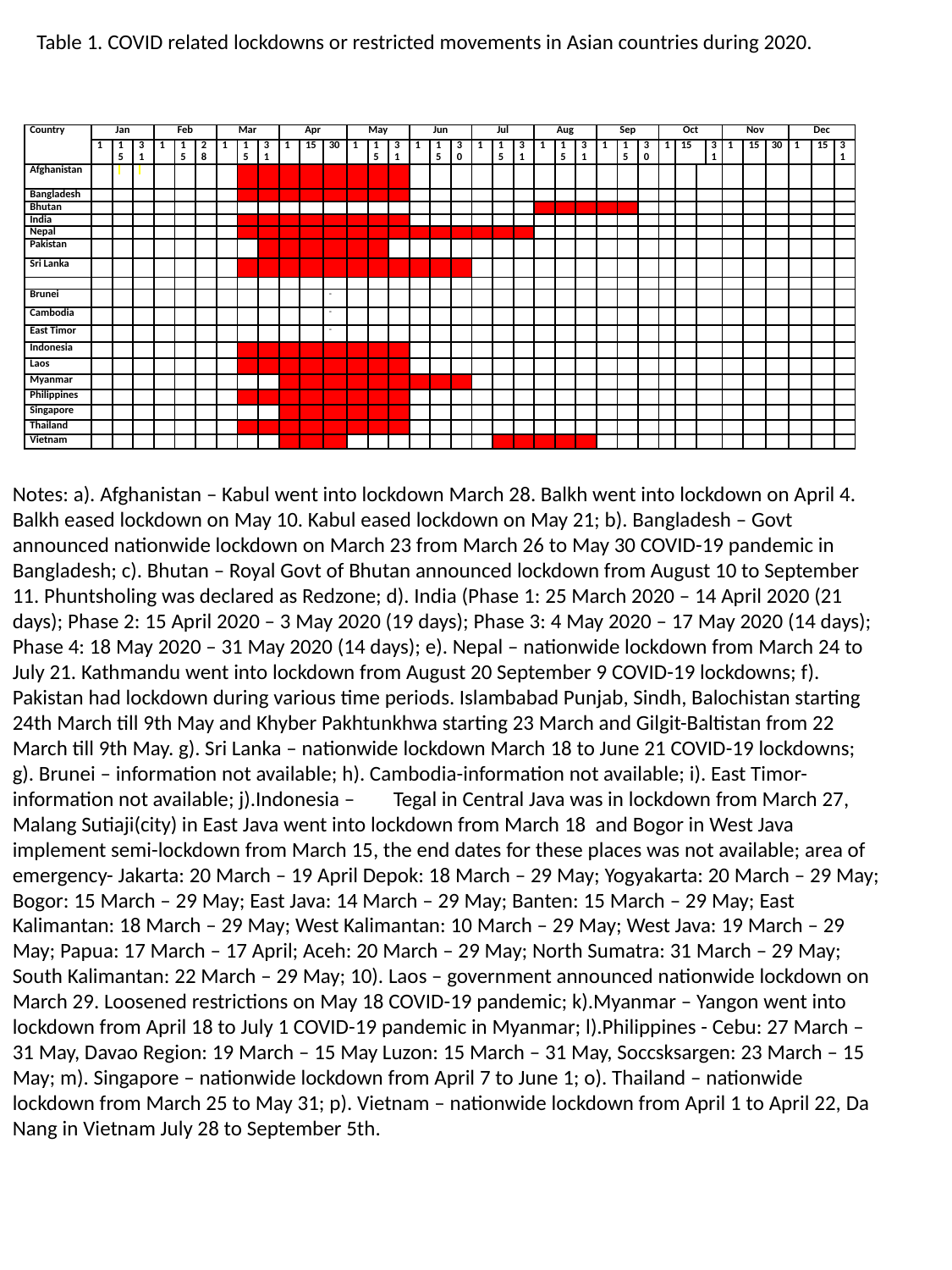

Table 1. COVID related lockdowns or restricted movements in Asian countries during 2020.
| Country | Jan | | | Feb | | | Mar | | | Apr | | | May | | | Jun | | | Jul | | | Aug | | | Sep | | | Oct | | | | Nov | | | Dec | | |
| --- | --- | --- | --- | --- | --- | --- | --- | --- | --- | --- | --- | --- | --- | --- | --- | --- | --- | --- | --- | --- | --- | --- | --- | --- | --- | --- | --- | --- | --- | --- | --- | --- | --- | --- | --- | --- | --- |
| | 1 | 15 | 31 | 1 | 15 | 28 | 1 | 15 | 31 | 1 | 15 | 30 | 1 | 15 | 31 | 1 | 15 | 30 | 1 | 15 | 31 | 1 | 15 | 31 | 1 | 15 | 30 | 1 | 15 | | 31 | 1 | 15 | 30 | 1 | 15 | 31 |
| Afghanistan | | | | | | | | | | | | | | | | | | | | | | | | | | | | | | | | | | | | | |
| Bangladesh | | | | | | | | | | | | | | | | | | | | | | | | | | | | | | | | | | | | | |
| Bhutan | | | | | | | | | | | | | | | | | | | | | | | | | | | | | | | | | | | | | |
| India | | | | | | | | | | | | | | | | | | | | | | | | | | | | | | | | | | | | | |
| Nepal | | | | | | | | | | | | | | | | | | | | | | | | | | | | | | | | | | | | | |
| Pakistan | | | | | | | | | | | | | | | | | | | | | | | | | | | | | | | | | | | | | |
| Sri Lanka | | | | | | | | | | | | | | | | | | | | | | | | | | | | | | | | | | | | | |
| | | | | | | | | | | | | | | | | | | | | | | | | | | | | | | | | | | | | | |
| Brunei | | | | | | | | | | | | - | | | | | | | | | | | | | | | | | | | | | | | | | |
| Cambodia | | | | | | | | | | | | - | | | | | | | | | | | | | | | | | | | | | | | | | |
| East Timor | | | | | | | | | | | | - | | | | | | | | | | | | | | | | | | | | | | | | | |
| Indonesia | | | | | | | | | | | | | | | | | | | | | | | | | | | | | | | | | | | | | |
| Laos | | | | | | | | | | | | | | | | | | | | | | | | | | | | | | | | | | | | | |
| Myanmar | | | | | | | | | | | | | | | | | | | | | | | | | | | | | | | | | | | | | |
| Philippines | | | | | | | | | | | | | | | | | | | | | | | | | | | | | | | | | | | | | |
| Singapore | | | | | | | | | | | | | | | | | | | | | | | | | | | | | | | | | | | | | |
| Thailand | | | | | | | | | | | | | | | | | | | | | | | | | | | | | | | | | | | | | |
| Vietnam | | | | | | | | | | | | | | | | | | | | | | | | | | | | | | | | | | | | | |
Notes: a). Afghanistan – Kabul went into lockdown March 28. Balkh went into lockdown on April 4. Balkh eased lockdown on May 10. Kabul eased lockdown on May 21; b). Bangladesh – Govt announced nationwide lockdown on March 23 from March 26 to May 30 COVID-19 pandemic in Bangladesh; c). Bhutan – Royal Govt of Bhutan announced lockdown from August 10 to September 11. Phuntsholing was declared as Redzone; d). India (Phase 1: 25 March 2020 – 14 April 2020 (21 days); Phase 2: 15 April 2020 – 3 May 2020 (19 days); Phase 3: 4 May 2020 – 17 May 2020 (14 days); Phase 4: 18 May 2020 – 31 May 2020 (14 days); e). Nepal – nationwide lockdown from March 24 to July 21. Kathmandu went into lockdown from August 20 September 9 COVID-19 lockdowns; f). Pakistan had lockdown during various time periods. Islambabad Punjab, Sindh, Balochistan starting 24th March till 9th May and Khyber Pakhtunkhwa starting 23 March and Gilgit-Baltistan from 22 March till 9th May. g). Sri Lanka – nationwide lockdown March 18 to June 21 COVID-19 lockdowns; g). Brunei – information not available; h). Cambodia-information not available; i). East Timor-information not available; j).Indonesia –	Tegal in Central Java was in lockdown from March 27, Malang Sutiaji(city) in East Java went into lockdown from March 18 and Bogor in West Java implement semi-lockdown from March 15, the end dates for these places was not available; area of emergency- Jakarta: 20 March – 19 April Depok: 18 March – 29 May; Yogyakarta: 20 March – 29 May; Bogor: 15 March – 29 May; East Java: 14 March – 29 May; Banten: 15 March – 29 May; East Kalimantan: 18 March – 29 May; West Kalimantan: 10 March – 29 May; West Java: 19 March – 29 May; Papua: 17 March – 17 April; Aceh: 20 March – 29 May; North Sumatra: 31 March – 29 May; South Kalimantan: 22 March – 29 May; 10). Laos – government announced nationwide lockdown on March 29. Loosened restrictions on May 18 COVID-19 pandemic; k).Myanmar – Yangon went into lockdown from April 18 to July 1 COVID-19 pandemic in Myanmar; l).Philippines - Cebu: 27 March – 31 May, Davao Region: 19 March – 15 May Luzon: 15 March – 31 May, Soccsksargen: 23 March – 15 May; m). Singapore – nationwide lockdown from April 7 to June 1; o). Thailand – nationwide lockdown from March 25 to May 31; p). Vietnam – nationwide lockdown from April 1 to April 22, Da Nang in Vietnam July 28 to September 5th.

## Slide 3
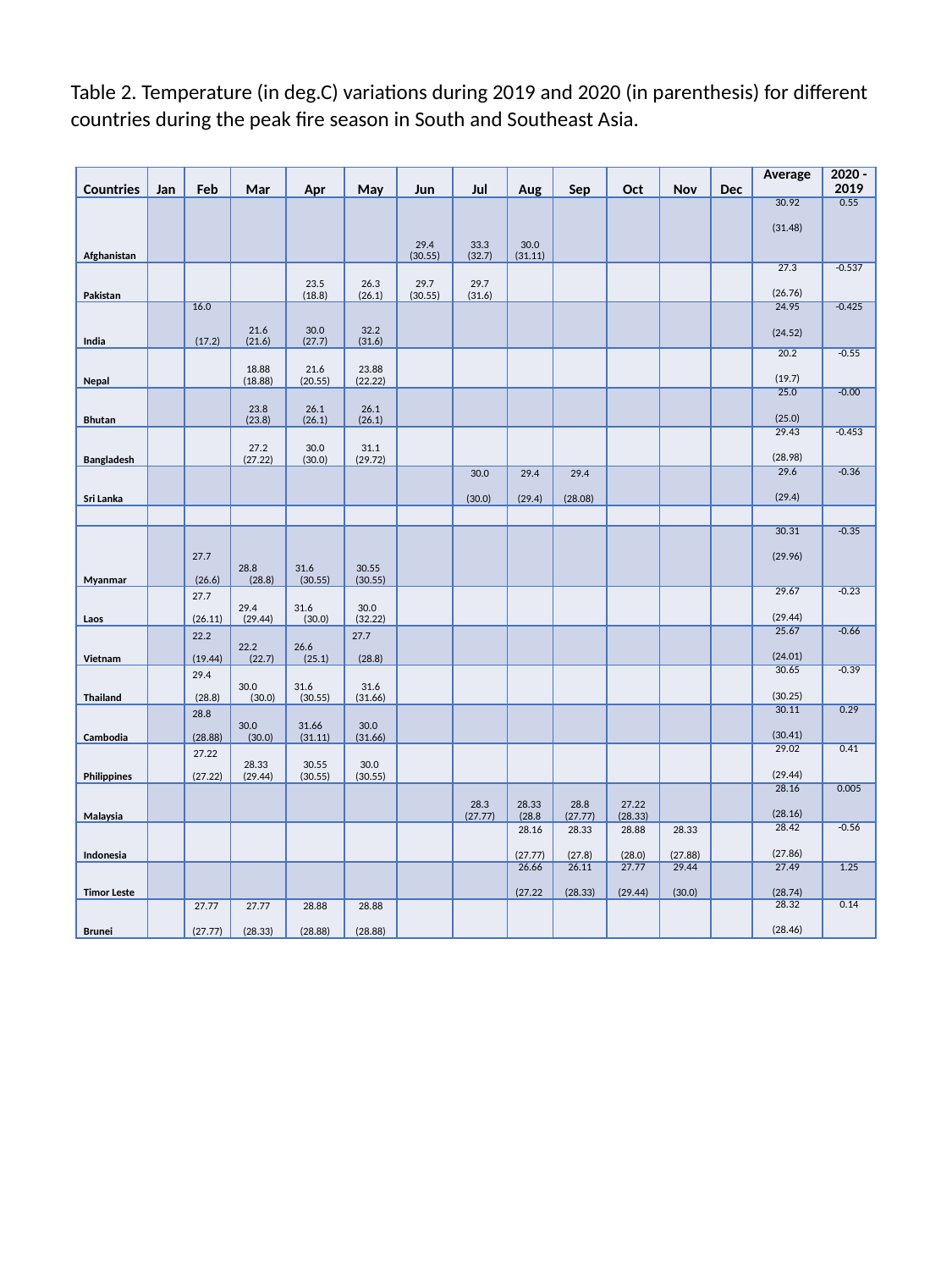

Table 2. Temperature (in deg.C) variations during 2019 and 2020 (in parenthesis) for different countries during the peak fire season in South and Southeast Asia.
| Countries | Jan | Feb | Mar | Apr | May | Jun | Jul | Aug | Sep | Oct | Nov | Dec | Average | 2020 -2019 |
| --- | --- | --- | --- | --- | --- | --- | --- | --- | --- | --- | --- | --- | --- | --- |
| Afghanistan | | | | | | 29.4 (30.55) | 33.3 (32.7) | 30.0 (31.11) | | | | | 30.92 (31.48) | 0.55 |
| Pakistan | | | | 23.5 (18.8) | 26.3 (26.1) | 29.7 (30.55) | 29.7 (31.6) | | | | | | 27.3 (26.76) | -0.537 |
| India | | 16.0 (17.2) | 21.6 (21.6) | 30.0 (27.7) | 32.2 (31.6) | | | | | | | | 24.95 (24.52) | -0.425 |
| Nepal | | | 18.88 (18.88) | 21.6 (20.55) | 23.88 (22.22) | | | | | | | | 20.2 (19.7) | -0.55 |
| Bhutan | | | 23.8 (23.8) | 26.1 (26.1) | 26.1 (26.1) | | | | | | | | 25.0 (25.0) | -0.00 |
| Bangladesh | | | 27.2 (27.22) | 30.0 (30.0) | 31.1 (29.72) | | | | | | | | 29.43 (28.98) | -0.453 |
| Sri Lanka | | | | | | | 30.0 (30.0) | 29.4 (29.4) | 29.4 (28.08) | | | | 29.6 (29.4) | -0.36 |
| | | | | | | | | | | | | | | |
| Myanmar | | 27.7 (26.6) | 28.8 (28.8) | 31.6 (30.55) | 30.55 (30.55) | | | | | | | | 30.31 (29.96) | -0.35 |
| Laos | | 27.7 (26.11) | 29.4 (29.44) | 31.6 (30.0) | 30.0 (32.22) | | | | | | | | 29.67 (29.44) | -0.23 |
| Vietnam | | 22.2 (19.44) | 22.2 (22.7) | 26.6 (25.1) | 27.7 (28.8) | | | | | | | | 25.67 (24.01) | -0.66 |
| Thailand | | 29.4 (28.8) | 30.0 (30.0) | 31.6 (30.55) | 31.6 (31.66) | | | | | | | | 30.65 (30.25) | -0.39 |
| Cambodia | | 28.8 (28.88) | 30.0 (30.0) | 31.66 (31.11) | 30.0 (31.66) | | | | | | | | 30.11 (30.41) | 0.29 |
| Philippines | | 27.22 (27.22) | 28.33 (29.44) | 30.55 (30.55) | 30.0 (30.55) | | | | | | | | 29.02 (29.44) | 0.41 |
| Malaysia | | | | | | | 28.3 (27.77) | 28.33 (28.8 | 28.8 (27.77) | 27.22 (28.33) | | | 28.16 (28.16) | 0.005 |
| Indonesia | | | | | | | | 28.16 (27.77) | 28.33 (27.8) | 28.88 (28.0) | 28.33 (27.88) | | 28.42 (27.86) | -0.56 |
| Timor Leste | | | | | | | | 26.66 (27.22 | 26.11 (28.33) | 27.77 (29.44) | 29.44 (30.0) | | 27.49 (28.74) | 1.25 |
| Brunei | | 27.77 (27.77) | 27.77 (28.33) | 28.88 (28.88) | 28.88 (28.88) | | | | | | | | 28.32 (28.46) | 0.14 |

## Slide 4
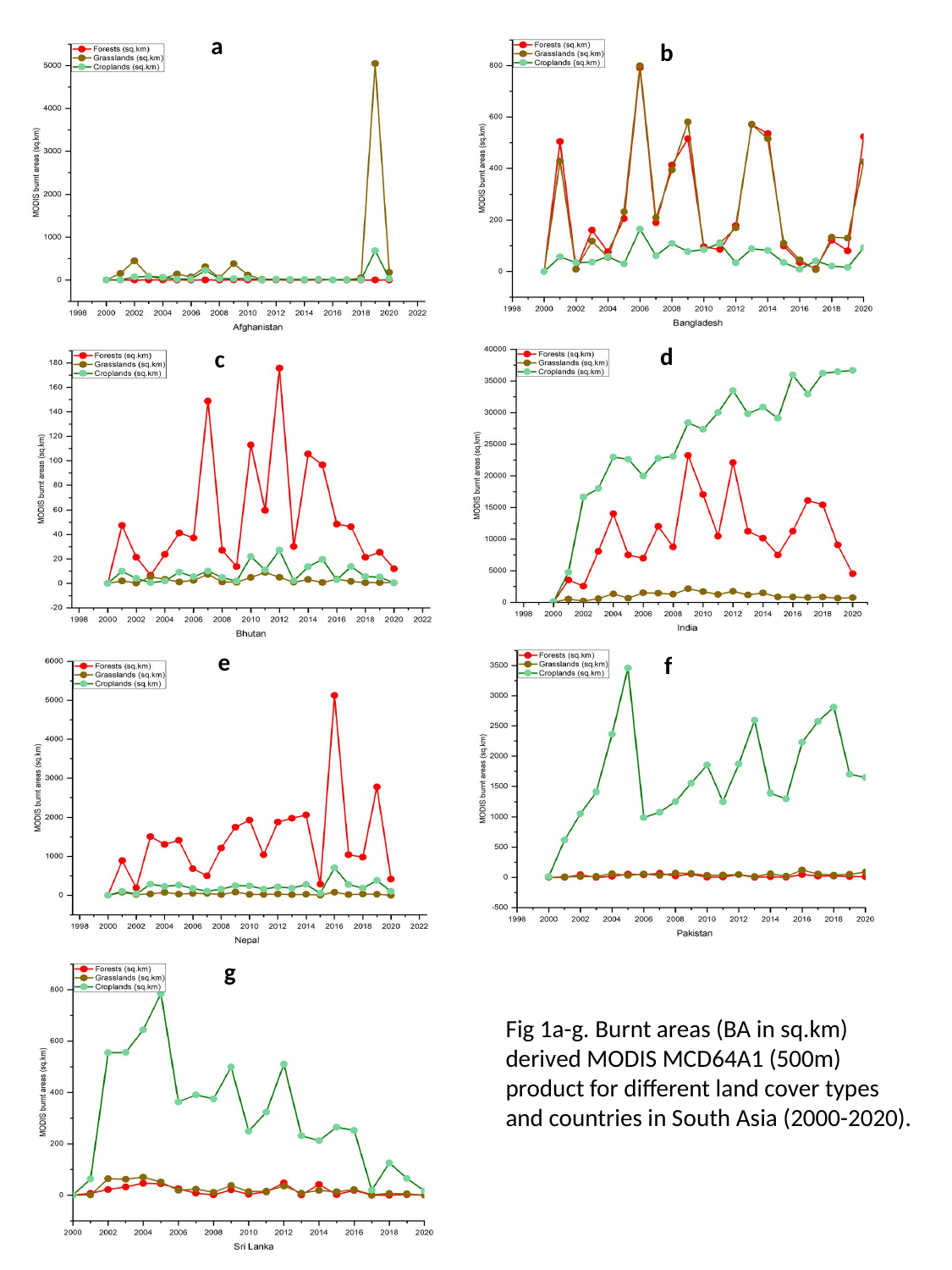

a
b
d
c
e
f
g
Fig 1a-g. Burnt areas (BA in sq.km) derived MODIS MCD64A1 (500m) product for different land cover types and countries in South Asia (2000-2020).

## Slide 5
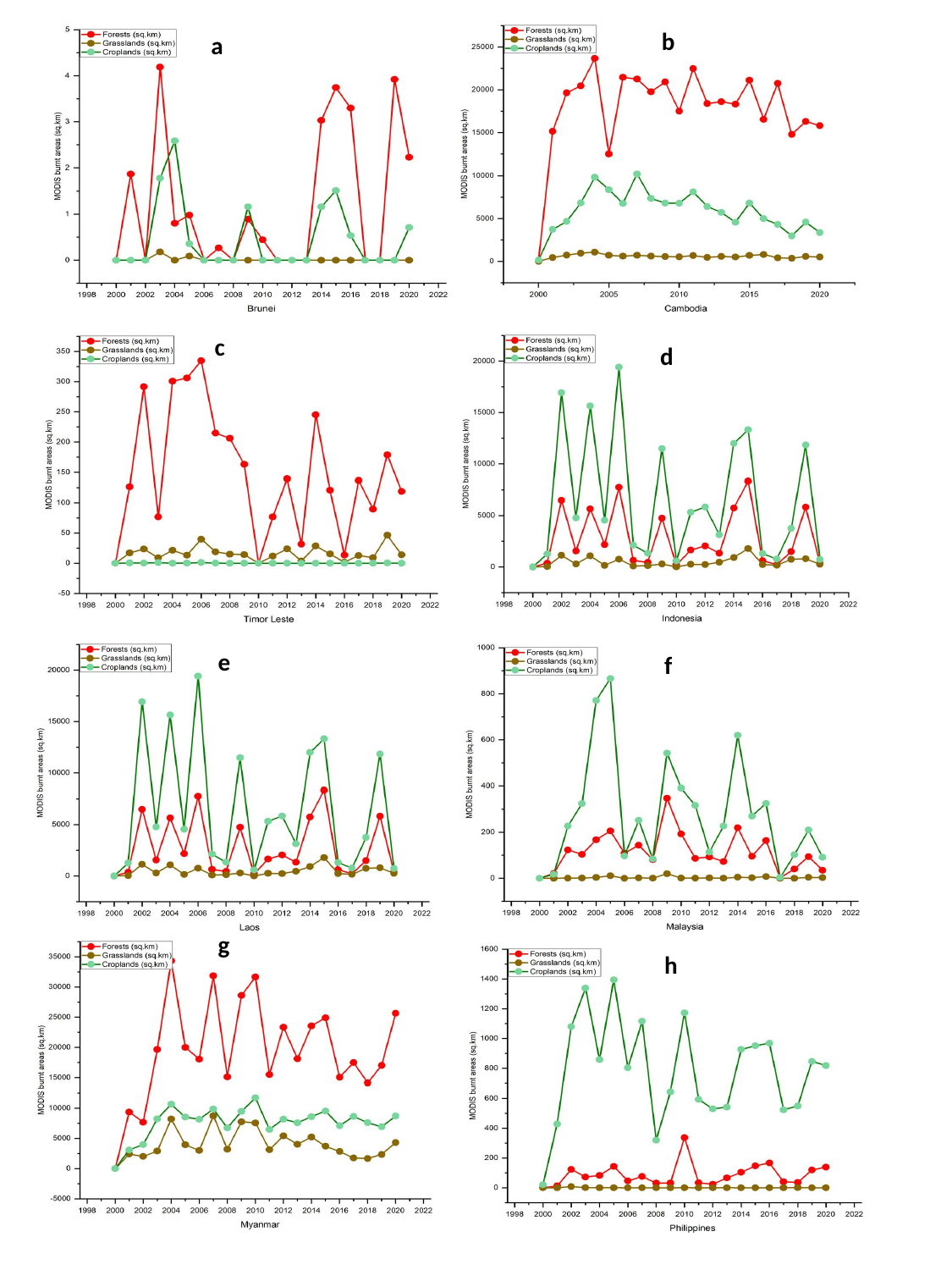

b
a
c
d
e
f
g
h

## Slide 6
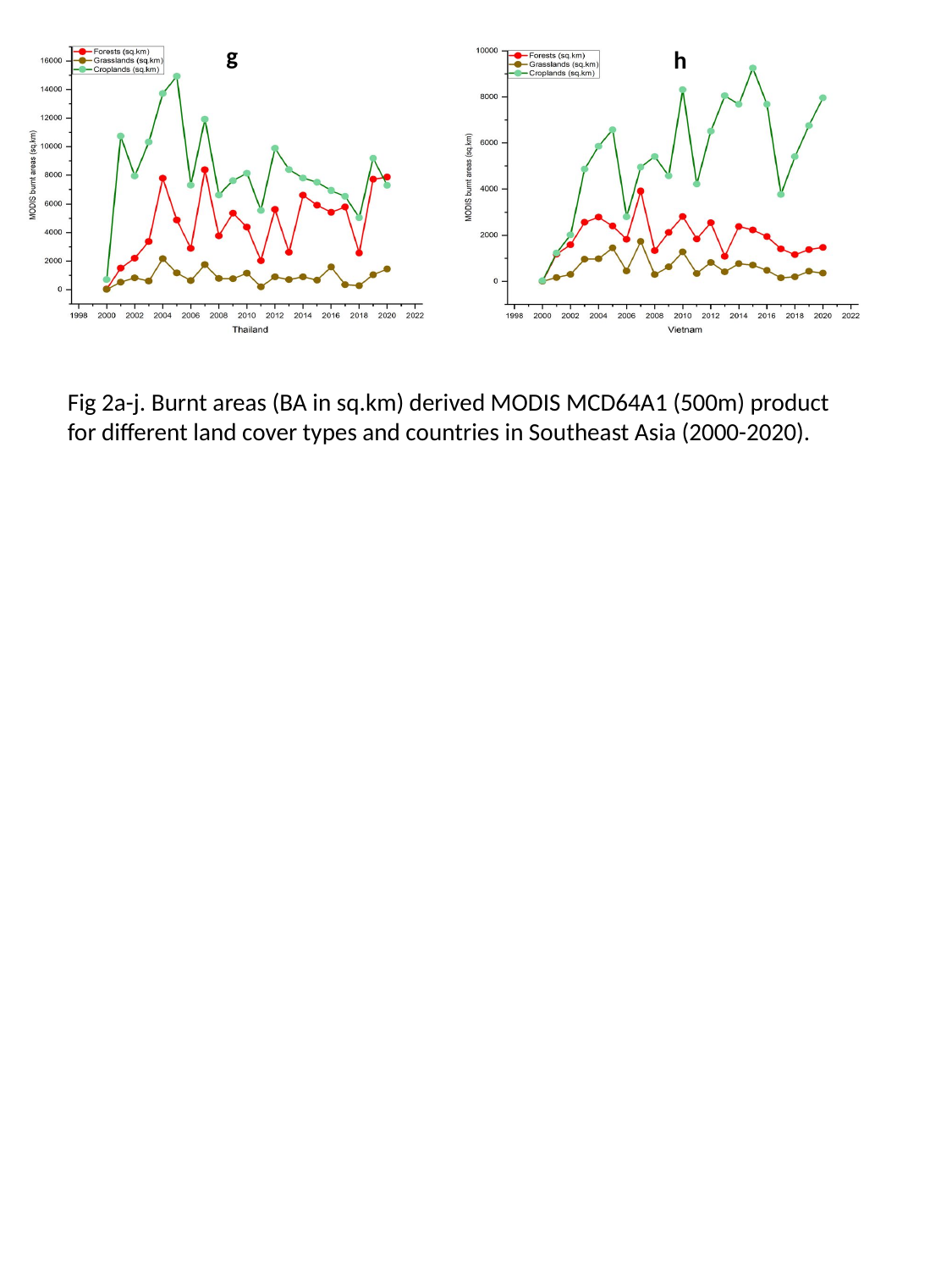

g
h
Fig 2a-j. Burnt areas (BA in sq.km) derived MODIS MCD64A1 (500m) product for different land cover types and countries in Southeast Asia (2000-2020).
